# The natural product carolacton inhibits folate-dependent C1 metabolism by targeting FolD/MTHFD

**DOI:** 10.1038/s41467-017-01671-5

**Published:** 2017-11-16

**Authors:** Chengzhang Fu, Asfandyar Sikandar, Jannik Donner, Nestor Zaburannyi, Jennifer Herrmann, Michael Reck, Irene Wagner-Döbler, Jesko Koehnke, Rolf Müller

**Affiliations:** 1 Department of Microbial Natural Products, Helmholtz Centre for Infection Research and Department of Pharmaceutical Biotechnology, Helmholtz Institute for Pharmaceutical Research Saarland (HIPS), Saarland University, Campus E8.1, 66123 Saarbrücken, Germany; 2 Helmholtz Institute for Pharmaceutical Research Saarland, Workgroup Structural Biology of Biosynthetic Enzymes, Helmholtz Centre for Infection Research, Saarland University, Campus E8.1, 66123 Saarbrücken, Germany; 3 Helmholtz Center for Infection Research (HZI), Group Microbial Communication, 38124 Braunschweig, Germany; 4 German Centre for Infection Research (DZIF), Partner Site Hannover, 38124 Braunschweig, Germany

## Abstract

The natural product carolacton is a macrolide keto-carboxylic acid produced by the myxobacterium *Sorangium cellulosum*, and was originally described as an antibacterial compound. Here we show that carolacton targets FolD, a key enzyme from the folate-dependent C1 metabolism. We characterize the interaction between bacterial FolD and carolacton biophysically, structurally and biochemically. Carolacton binds FolD with nanomolar affinity, and the crystal structure of the FolD–carolacton complex reveals the mode of binding. We show that the human FolD orthologs, MTHFD1 and MTHFD2, are also inhibited in the low nM range, and that micromolar concentrations of carolacton inhibit the growth of cancer cell lines. As mitochondrial MTHFD2 is known to be upregulated in cancer cells, it may be possible to use carolacton as an inhibitor tool compound to assess MTHFD2 as an anti-cancer target.

## Abstract (web summary)

The mechanisms behind the antibacterial activity of the natural product carolacton are unknown. Here, the authors show that carolacton is a potent inhibitor of FolD/MTHFD enzymes (involved in folate-dependent C1 metabolism in bacteria and humans) and inhibits the growth of cancer cell lines

## Introduction

Natural products have served as a source of novel drug leads for thousands of years, but for many of them their mechanism of action (MoA) remains unknown^1^. The secondary metabolite carolacton is a macrolide keto-carboxylic acid produced by the myxobacterium *Sorangium cellulosum* with an elusive molecular target^2^. Carolacton is a potent inhibitor of biofilm formation in the human pathogen *Streptococcus mutans* and a growth inhibitor of *S. pneumoniae* ^3^. It also inhibits the efflux pump mutant *Escherichia coli*Δ*tolC* ^2, 4^. Carolacton has moderate activity on various fungi but showed no acute toxicity to L929 mouse cells^2, 4^. Although carolacton seemed to be a very narrow spectrum antibiotic, its interesting anti-biofilm and anti-fungal activity aroused our interest, and we aimed to uncover its MoA.

**Fig. 1:**
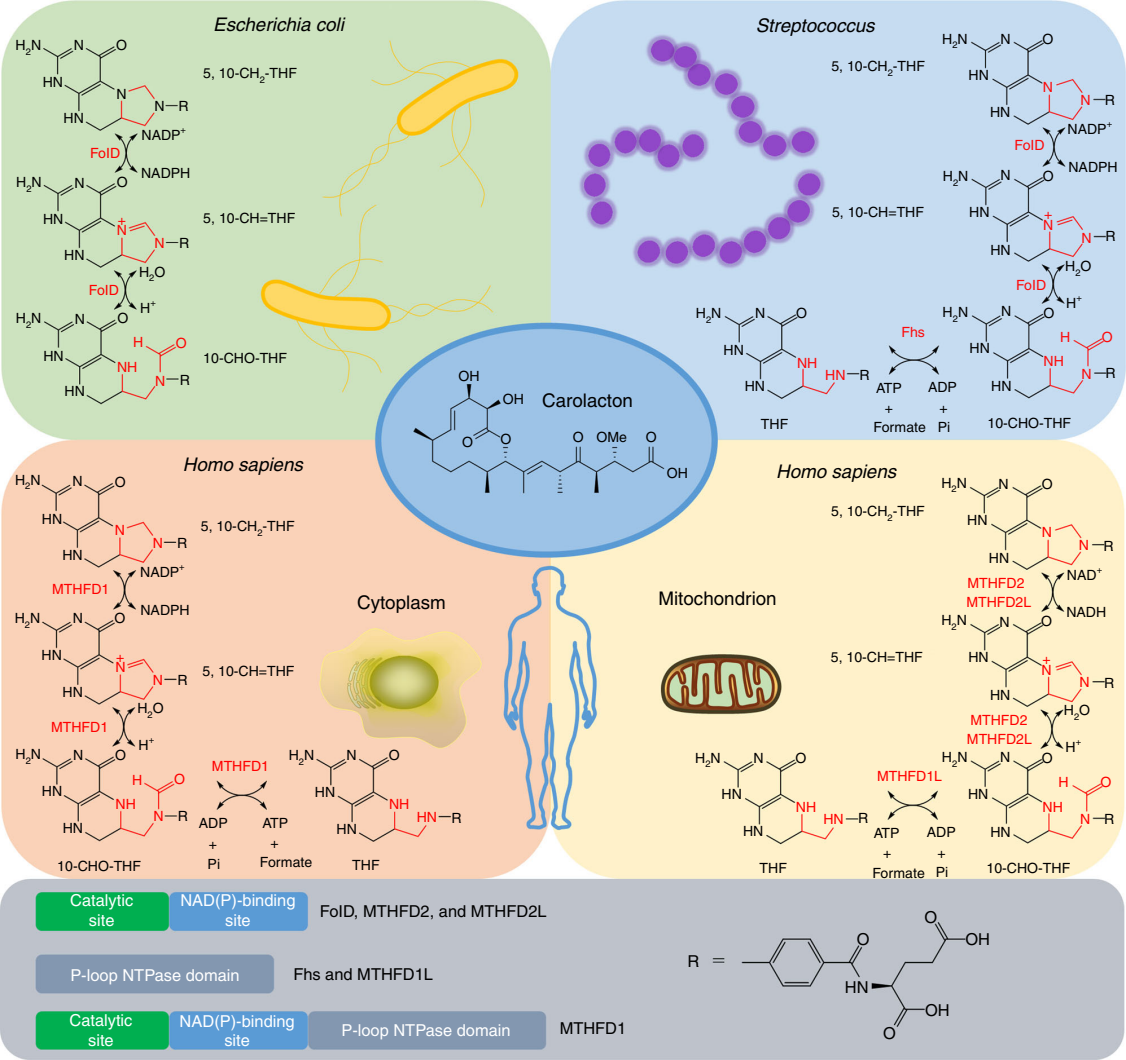
The enzymatic routes to synthesize 10-CHO-THF in different organisms. In *E. coli*, 10-CHO-THF is synthesized by FolD, while in several other bacteria such as Streptococci, 10-CHO-THF can be produced via an alternative enzyme named Fhs. In humans, there are different enzymes in the cytoplasm and the mitochondrion. In the cytoplasm, MTHFD1, which is a tri-functional enzyme, exerts FolD-DH, FolD-CYH and Fhs functions. In the mitochondrion, while MTHFD2 and MTHFD2L have FolD functions, MTHFD1L plays the same role as Fhs. The chemical structure of FolD inhibitor carolacton is shown in the centre

Here we report the identification and validation of FolD as the carolacton target in *E. coli*Δ*tolC* and streptococci. FolD occupies a central position in the folate-dependent C1 metabolism. Folate (vitamin B9) is an essential co-factor in all cells, but it is synthesized only by bacteria and plants^5^. The folate-dependent C1 metabolism is highly conserved in all domains of life and it provides the key building blocks for growth, most importantly nucleic acids, amino acids, provitamines and formylated methionine tRNA for translation initiation^6^. FolD is a dual function enzyme: it catalyses a reversible NADP^+^-dependent dehydrogenation step (5,10-methylenetetrahydrofolate (5,10-CH_2_-THF) dehydrogenase (DH)) and a subsequent cyclohydrolysis step (5,10-methenyltetrahydrofolate (5,10-CH=THF) cyclohydrolase (CYH)) (Fig. 1)^7^. We demonstrate that carolacton inhibits both reactions catalyzed by FolD at nM concentrations. Determination of the FolD/carolacton complex crystal structure allows us to rationalize how mutations in FolD can confer resistance to carolacton.

Higher organisms such as humans also possess FolD orthologs: the cytosolic trifunctional protein MTHFD1, which has DH and CYH, but also a formate tetrahydrofolate synthase (Fhs) domain, maintains the metabolism of methylene-, methenyl- and formyl-THF (10-CHO-THF)^8–10^. In mitochondria, the DH and CYH activities are provided by the FolD analogues MTHFD2 and MTHFD2L (Fig. 1)^9, 10^. The enzymes involved in folate-dependent C1 metabolism, therefore, represent powerful targets for the inhibition of fast growing cells and have been targeted by anticancer drugs such as clinically used methotrexate, which inhibits dihydrofolate reductase^11^. Because of its central role in the C1 metabolism, FolD was the subject of numerous studies: Although synthetic folate-analogue inhibitors were found that strongly inhibited FolD in vitro, none were active on whole bacterial cells^12–15^. Several studies have reported inhibitors of the FolD analogues MTHFD1 or MTHFD2, some of which display in vivo activity against human cells^15–17^. However, either poor activity was observed (e.g. LY345899, IC_50_ 128 μM) or the compounds were unspecific (e.g. LY231514, which principally inhibits thymidylate synthase) (Supplementary Fig. 1)^16, 18, 19^.

To gauge carolacton’s potential for inhibiting the human FolD variants, we test it against the two human FolD orthologs, MTHFD1 and MTHFD2, which are also inhibited in the low nM range. When we tested carolacton’s effects on a panel of human cell lines, we observed activity in the µM range. Since MTHFD2 is differentially upregulated in cancer tissue, it presents an attractive target for anti-cancer compounds. We believe that carolacton, a non-substrate inhibitor, may be used as a tool compound to assess MTHFD2 as an anti-cancer target^20, 21^.

## Results

### FolD is the carolacton target

**Fig. 2:**
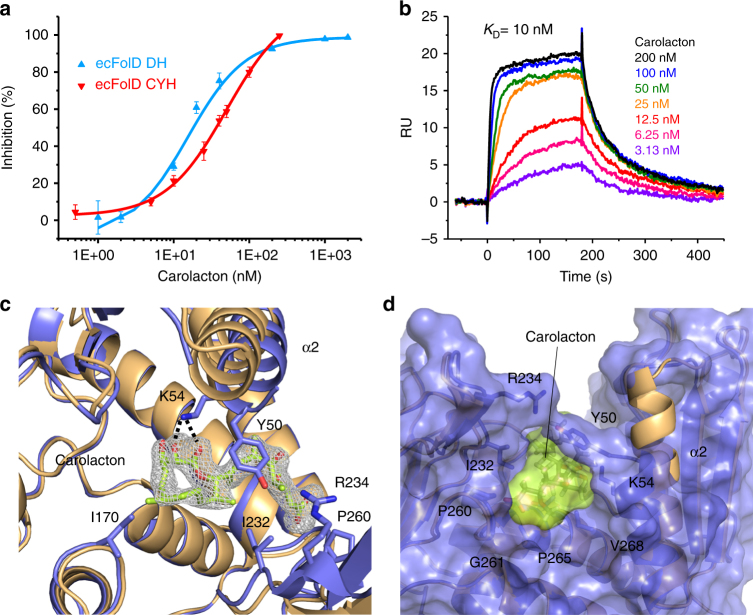
Carolacton is a potent and tightly binding inhibitor of FolD. **a** IC_50_ determination for carolacton against DH and CYH activities of ecFolD. Data are presented as means ± s.e.m of three independent replicates. IC_50_s were obtained via logistic dose–response fitting. The one-way ANOVA test was used for statistical analysis, *P* < 0.01. **b** SPR analysis of carolacton binding to ecFolD, RU resonance units. Carolacton shows strong binding to ecFolD. The estimated *K*_D_ was obtained by fitting the association and dissociation signals with a 1:1 interacton model using the Biacore X100 Evaluation Software. **c** Top view of the FolD–carolacton complex structure. Cartoon representations of apo-FolD (gold) and FolD in complex with carolacton (blue, lime) are superposed to show the movement and partial dissolution of helix α2. Residues involved in carolacton binding are shown as sticks. The difference electron density (*F*_o_−*F*_c_) contoured to 3*σ* with phases calculated from a model that was refined in the absence of carolacton is shown as a grey isomesh. **d** Surface representation of FolD bound to carolacton, side-view. Colours correspond to **c**. Partial dissolution of helix α2 as a result of the interaction of Y50, which forms a lid on carolacton that is further stabilized by R234, can be seen

To facilitate the identification of the molecular target of carolacton, *E. coli*Δ*tolC* (*E. coli* without outer membrane efflux protein TolC) was chosen to develop carolacton-resistant mutants. After 1 week, carolacton-resistant mutants arose spontaneously on agar plates supplemented with four times the carolacton minimum inhibitory concentration (MIC = 0.125 μg/mL). Whole-genome sequencing of five independent mutants unveiled that only the gene encoding FolD was mutated (Supplementary Tables 1 and 2). Four different mutations conferring resistance were identified: G8S (observed in two mutants), K54N, Q98H and K54_K56delinsK (hereafter referred to as ΔK54R55) (Supplementary Figs. 2 and 3). To validate FolD as the carolacton target and to understand the potential MoA, *E. coli*Δ*tolC* FolD (ecFolD) was overexpressed and purified. A SDS-PAGE of all proteins used in this study, as well as their LC-MS analysis, can be found in Supplementary Fig. 4. The purified ecFolD showed DH and CYH activity comparable to previously reported bacterial FolDs^13, 22, 23^, including the DH activity of *E. coli* FolD^24^. DH and CYH activities were found to be much stronger than reported for ecFolD in two other studies^25, 26^ (Supplementary Table 3). This discrepancy has been noted by other authors and possible reasons include impurities^24, 26^ and excessive enzyme concentrations used for enzyme kinetics^25^. When carolacton was added to the reaction, we observed strong inhibition of both steps catalysed by ecFolD (DH and CYH) in a carolacton concentration-dependent manner (Fig. 2a). Carolacton also showed competitive inhibition with both substrates and the cofactor involved in DH and CYH catalytic steps, which were 5,10-CH_2_-THF (*K*_*i*_ = 21 nM), NADP^+^ (*K*_*i*_ = 11 nM) and 5,10-CH=THF (*K*_*i*_ = 32 nM) (Supplementary Table 4), as the apparent Michaelis constant (*K*_M_) increased when carolacton was added (Supplementary Fig. 5). These findings supported that ecFolD is indeed the target of carolacton. To confirm that both molecules interact, we then tested ecFolD for its ability to bind carolacton using surface plasmon resonance (SPR). The observed interaction between ecFolD and carolacton was very strong (*K*_D_ = 10 nM), further confirming ecFolD as the carolacton target (Fig. 2b and Supplementary Table 5).

### The structure of the ecFolD–carolacton complex

To understand how carolacton binds to ecFolD and thus rationalize the mutations giving rise to carolacton resistance, we determined the crystal structure of ecFolD in complex with carolacton. The originally reported crystallization conditions for ecFolD^27^ yielded only poorly diffracting crystals in our hands. Reductive lysine methylation ecFolD^Meth^ gave a new crystal form that diffracted well. Since a key active-site residue of ecFolD is a lysine (K54)^15^, and we found that mutations at this position conferred carolacton resistance (see resistant mutants above), we thought that K54 could be involved in carolacton binding, and thus sought to protect this residue from methylation. To this end, we incubated ecFolD with an excess of carolacton before lysine methylation. After the reaction, carolacton was removed via size exclusion chromatography and the resultant ecFolD^Meth^ tested for activity. We observed only a slight decrease in enzyme activity (Supplementary Fig. 6a) and no significant change of carolacton’s ability to inhibit ecFolD DH activity (Supplementary Fig. 6b). To further analyse whether we had methylated active-site K54, we first determined the apo-structure of ecFolD^Meth^. The protein crystallized in space group P2_1_ and crystals diffracted to 1.9 Å resolution (PDB ID 5o28). Full data collection and refinement statistics for all structures can be found in Supplementary Table 6. ecFolD^Meth^ showed the canonical dimeric arrangement observed in the structures of ecFolD (PDB ID 1B0A)^27^ and its orthologs^17, 28^ and the overall structure was virtually unchanged when compared to unmethylated protein (Supplementary Fig. 7). We observed unambiguous electron density for methylated lysines at positions K4, 22, 194, 212 and 222. With the exception of K22, all of them can be found at crystal contacts (Supplementary Fig. 8). We observed density for unmethylated active-site K54, supporting our biochemical data that indicated this lysine was not methylated (Supplementary Figs. 6 and 8). We soaked the apo crystals of ecFolD^Meth^ with 2 mM carolacton and harvested them after 16 h. The soaked crystals diffracted to 2.1 Å (PDB ID 5o22) and showed strong additional density at the protein’s active site in three of the four monomers of the asymmetric unit (protomers A, B and D, the picture in protomer C is skewed due to a crystal contact). Carolacton can be fitted into the additional density (Fig. 2c) and refines well (Supplementary Fig. 8). Binding of carolacton to FolD has a minimal impact on FolD’s overall structure (Cα rmsd 0.27 Å) and is facilitated by three hydrogen bonds (K54 ε-amino group with carolacton O8 and O33 and G261 main chain N with carolacton O29) and several hydrophobic interactions (Y50, I170, I232, P260, P265 and V268) (Fig. 2c, d and Supplementary Fig. 9). The hydrophobic interaction between carolacton and Y50 causes a partial disruption of helix α2 (residues 45–56) and allows the Y50 hydroxyl group to form a hydrogen bond with the side-chain of R234 (Fig. 2d). When carolacton is bound, the N-terminal residues of α2 and a part of the loop connecting β1 with α2 (residues 41–44) become disordered (residues 44–49). It has been noted by others that this part of FolD is unstable^15, 17^, and we observe only poor density for residues 44–56 in the ecFolD^Meth^ apo structure (protomers A, B and D; protomer C behaves differently due to a crystal contact). We believe that this reflects a catalytic mechanism, in which a meta-stable helix (α2) harbours a residue essential for catalysis. Upon substrate (or carolacton) binding, Y50 engages in hydrophobic contact that completely destabilizes residues 44–49, while the other half of the helix is stabilized (residues 50–56) (Fig. 2d).

When the FolD–carolacton complex structure is superposed onto the human ortholog of FolD (PDB ID 1DIA), which was co-crystallized with NADP^+^, and substrate analogue LY249543, it can be seen that carolacton is likely to prevent binding of both co-factor and substrates (Supplementary Fig. 10a, b). In a recent study, a number of carolacton analogues were synthesized and tested for their effects on *S. mutans* biofilm formation. None of the reported compounds had improved bioactivity^29^. We modelled the best-performing compound (‘carylacton’, see structure in Supplementary Fig. 1) based on our complex structure and discovered that carylacton would be able to form better hydrophobic interactions with Y50 than carolacton. The length of the carylacton tail, however, is one methylene group too long to allow hydrogen bonding of the carboxy terminus with G261 (Supplementary Fig. 10c). Our complex structure should therefore help future synthetic efforts.

### Effects of mutations on ecFolD and carolacton binding

The effects of mutations K54N and G8S on carolacton binding can easily be explained by the complex crystal structure. K54 provides two hydrogen bonds to anchor carolacton in the active site of ecFolD (Fig. 2c) and its mutation to N will severely affect the binding of carolacton to ecFolD. G8 is not in direct contact with carolacton, but its position and orientation in FolD implies that the addition of any side-chain will cause a clash with C-terminal helix α11 (Supplementary Fig. 11). G261, which forms a hydrogen bond with carolacton, is part of this helix and we assume that the resulting movement of helix α11 leads to a clash with the carboxyl group of carolacton. Due to the very tight fit of carolacton in this area, the clash cannot be remedied, but instead leads to severely reduced binding of carolacton to ecFolD. Helix α2 is vital for binding of carolacton to FolD since it contains both K54 and Y50. It was therefore not unexpected that the deletion of two residues from this helix (mutant ecFolDΔK54R55) severely affected binding of carolacton to FolD. The final mutation, Q98H, could not be rationalized using the complex structure and we wondered if this mutation does in fact affect binding of carolacton to ecFolD or is an experimental artefact.

**Fig. 3:**
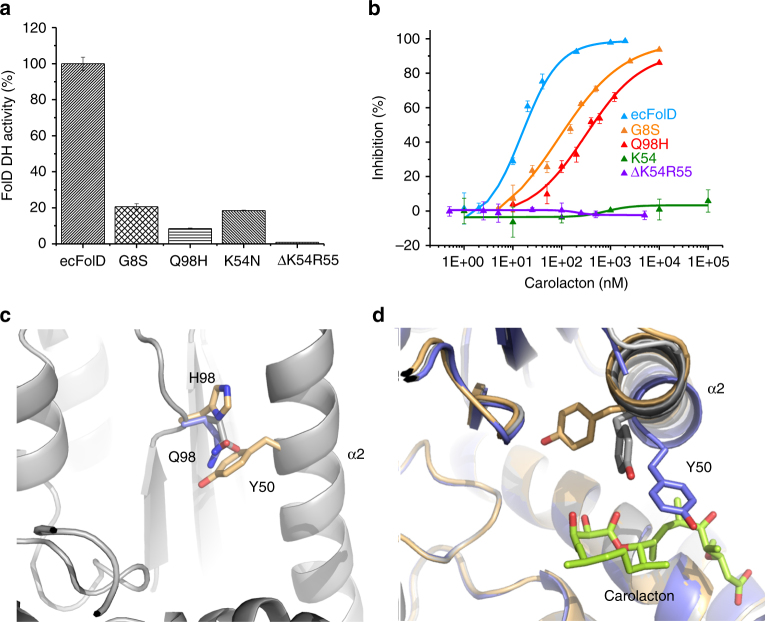
Effects of FolD mutations on DH activity and carolacton binding. **a** Residual DH activity of ecFolD mutants compared to ecFolD. Data are presented as means ± s.e.m of three independent replicates. DH specific activities were calculated based on the *V*_max_ obtained via Michaelis–Menten fitting. The one-way ANOVA test was used for statistical analysis, *P* < 0.01. **b** IC_50_ determination for carolacton against the DH activity of ecFolD and mutants. Data are presented as means ± s.e.m of three independent replicates. IC_50_s were obtained via logistic dose–response fitting. The one-way ANOVA test was used for statistical analysis, *P* < 0.01. **c** Position of Y50 in ecFolDQ98H (gold). The position of Q98 in the apo (grey) and complex (blue) structures prevents the side-chain of Y50 from rotating out of position. **d** Position of Y50 in the apo (grey), complex (blue) and Q98H (gold) structures. The 90° rotation of Y50 in the Q98H mutant prevents Y50 from forming the hydrophobic lid on carolacton (lime sticks)

To test experimentally the effects of the mutations that we discovered in carolacton resistant *E. coli* Δ*tolC*, we expressed and purified all four mutant proteins: ecFolDG8S, ecFolDK54N, ecFolDΔK54R55 and ecFolDQ98H. When we tested the mutants for DH activity, we found that all of them were attenuated, but with varying severity. The mutant K54N still retains ~19% of the wild-type (wt) DH activity, but ΔK54R55 has only ~1% DH activity left. The two-residue deletion might severely change the NADP^+^ binding, which can be deduced from the dramatically increased *K*_M_ of NADP^+^. The DH activity of G8S and Q98H is about 21 and 8% of the wt, respectively (Fig. 3a and Supplementary Table 7).

Mutants K54N and ΔK54R55 showed no detectable CYH activity, suggesting that K54 is essential for this reaction. The kinetic properties of the CYH activity of mutants G8S and Q98H were not investigated since only very low CYH activity was detected. We estimate residual activity of ~1% based on the requirement to increase the enzyme concentration ~100-fold to achieve wt turnover (Supplementary Fig. 12). The mutant *E. coli* Δ*tolC* strains likely survive without CYH activity because the turnover of 5,10-CH=THF to 10-CHO-THF can occur spontaneously^30^. These biochemical results are consistent with the growth curves measured for these carolacton-resistant *E. coli* Δ*tolC* strains: the one bearing ΔK54R55 mutation is the poorest growing mutant. The K54N mutant strain also grows very slowly even though its DH activity is not impaired as much as in the ΔK54R55 mutant, which might be due to the total loss of CYH activity (Supplementary Fig. 13).

The mutants G8S and Q98H could still be inhibited by carolacton, but with much higher IC_50_ compared to the wt (Fig. 3b and Supplementary Table 8). The central role of K54 is highlighted by the fact that the residual DH activity of K54N and ΔK54R55 is completely insensitive to carolacton (Fig. 3b), in accordance with the SPR data that indicated that carolacton does not bind to K54N (Supplementary Fig. 14a). It appears that, under selection pressure, viable mutants had sacrificed CYH activity (reaction can be spontaneous), but required retention of FolD’s DH activity since no alternative pathway exists in *E. coli* ^31^.

The binding of carolacton to ecFolD Q98H was seriously attenuated as reflected by the SPR data (Supplementary Fig. 14b, c). To understand how the mutation Q98H affects the binding of carolacton, we determined the crystal structure of this mutant (PDB ID 5o2a). After lysine methylation, this protein crystallized in the same space group as the wt protein and crystals diffracted to 1.9 Å. The overall structure of ecFolD^Meth^Q98H is not altered when compared to wt as a result of the mutation (Cα rmsd 0.11 Å) and the effect of the Q98H mutation was unexpected. The presence of H98 allows the side-chain of Y50 to rotate 90° compared to the apo wt ecFolD structure. It appears that in the wt protein, Q98 helps to position the side-chain of Y50 such that it can easily rotate to engage in hydrophobic interactions with carolacton (or substrate)  upon binding. In Q98H, the side-chain of Y50 is able to rotate farther from the active site and packs against residues V38, L40, L96 and H98. The result is a fully ordered helix α2 with good density for all residues, which we believe reflects a stabilization of the protein. The absence of the hydrophobic interaction between carolacton and Y50 leads to significantly weakened binding, which we believe to be reflected by the much faster association and dissociation of carolacton in SPR experiments with ecFolDQ98H (Fig. 3c, d, Supplementary Fig. 14b and Supplementary Table 5).

### Effects of carolacton on *S. pneumoniae* FolD

Carolacton was originally discovered as an anti-streptococcal compound^2, 4^, and the FolD ortholog from *S. pneumoniae* (spFolD) shares 49% sequence identity with ecFolD. The key residues involved in carolacton binding are conserved, and carolacton binds tightly to spFolD (*K*_*D*_ = 27 nM) (Supplementary Fig. 14d and Supplementary Table 5). Carolacton also showed competitive inhibition of spFolD with slightly higher inhibition constants than ecFolD, which were still in the low nM range (Supplementary Figs. 15 and 16 and Supplementary Tables 4 and 8). Analysis of the inhibition of FolD in streptococci is complicated by the fact that they, unlike *E. coli*, possess an alternative route for one-carbon metabolism via Fhs (Fig. 1)^31, 32^.

### Effects of carolacton on the human FolD orthologs

**Fig. 4:**
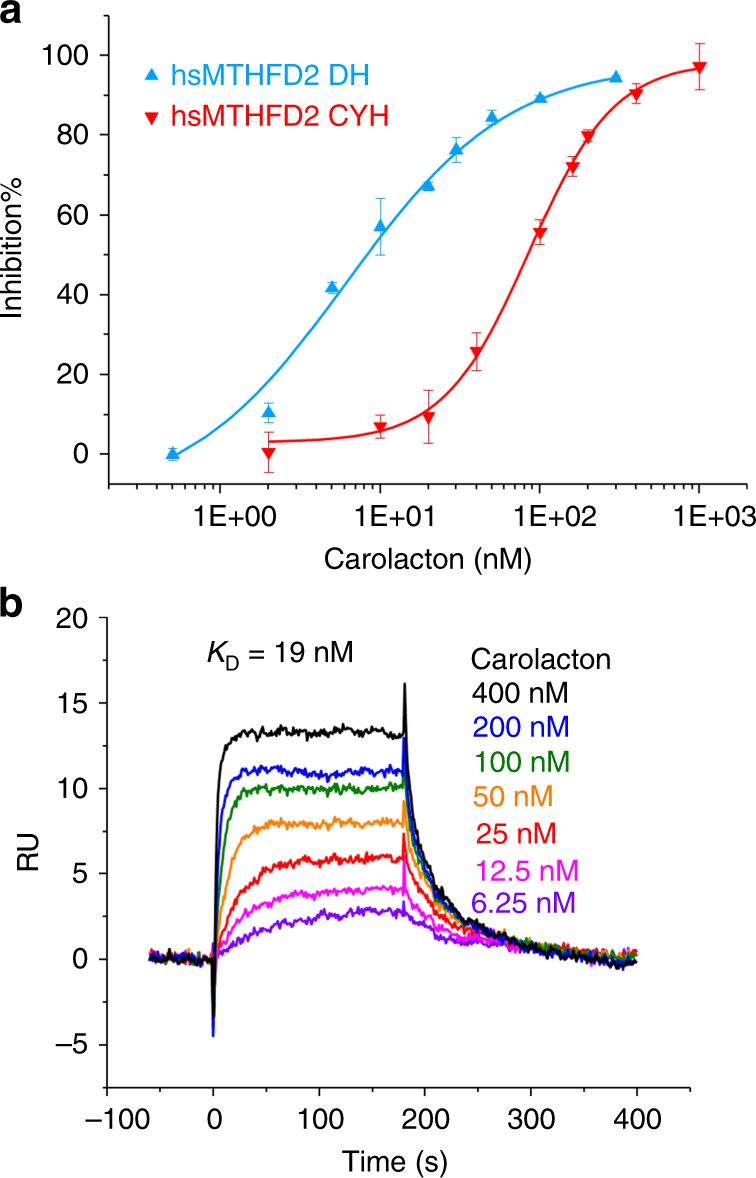
Carolacton effects on the human enzyme hsMTHFD2. **a** IC_50_ determination for carolacton against DH and CYH activity of hsMTHFD2. Data are presented as means ± s.e.m. of three independent replicates. IC_50_s were obtained via logistic dose–response fitting. The one-way ANOVA test was used for statistical analysis, *P* < 0.01. **b** SPR analysis of carolacton binding to hsMTHFD2. Carolacton shows strong binding to hsMTHFD2. The estimated *K*_D_ was obtained by fitting the association and dissociation signals with a 1:1 interacton model using the Biacore X 100 Evaluation Software

As mentioned above, FolD orthologs are also present in humans. The human enzymes hsMTHFD1_DC and hsMTHFD2 both share 42% sequence identity to ecFolD. The key residues involved in carolacton binding are fully conserved (Supplementary Fig. 3), and the structures of hsMTHFD1_DC and hsMTHFD2 are conserved well when compared to ecFolD (pairwise Cα rmsds of 1.09 and 0.84 Å, respectively, Supplementary Fig. 17). We were therefore confident that carolacton could also serve as an inhibitor of the human enzymes. This would be of particular interest since the mitochondrial hsMTHFD2 is overexpressed in many tumor cells and thus its inhibition might cause a selective effect on tumor growth^20^. We therefore expressed and purified hsMTHFD1_DC and hsMTHFD2 to investigate them biochemically. Not surprisingly, their inhibition by carolacton was similar to that observed for ecFolD (Fig. 4a and Supplementary Fig. 15). The *K*_*i*_ of carolacton against all three substrates and the cofactor were also determined and are in the same range as those for ecFolD (Supplementary Fig. 18 and Supplementary Tables 4 and 8). When the binding of carolacton to hsMTHFD2 was analysed by SPR, we found strong binding on par with the bacterial proteins (*K*_*D*_ = 19 nM) (Fig. 4b and Supplementary Table 5).

### Carolacton is active against human cancer cell lines

Since the human FolD orthologs are strongly inhibited by carolacton, we were curious to see if carolacton would show activity on human cancer cell lines. When tested against HCT-116, KB-3.1 and KB-V.1 cells, carolacton displayed an EC_50_ of 25, 11 and 42 µM, respectively. In each case, the maximum inhibition of cell growth was >80%. The inhibition of human epidermoid carcinoma KB-3.1 cells by carolacton is four times stronger than that of the corresponding multidrug-resistant (MDR) cell line KB-V.1 (Table 1). In the latter cell line, the *mdr1* gene is amplified, which encodes the drug transporter P-glycoprotein (Pgp); this suggests that Pgp may export carolacton from the cells. In the case of U-937 cells, carolacton displayed no activity at concentrations up to 100 µM. However, when this cell line is grown in folate-depleted medium, an EC_50_ of 42 µM and a maximum inhibition of 70% are observed. This apparent folate-dependent activity on U-937 cells supports that human FolD orthologs are the cellular targets of carolacton in human cancer cells.

**Table 1:** EC_50_ values of carolacton on human cancer cell lines

Cell line	EC_50_ (µM)	Maximum inhibition
HCT-116	24.6 ± 0.8	>80%
KB-3.1	11.3 ± 3.6	>80%
KB-V.1	41.8 ± 14.5	>80%
U-937	>100	n.a.
U-937 (folate-depleted)	41.7 ± 15.2	65–75%

### Efflux hampers carolacton activity

Carolacton shows no activity against wt *E. coli*, but is highly active against efflux protein-deficient *E. coli* Δ*tolC*. It also showed a four-fold decrease in activity against Pgp-overexpressing KB-V.1 cells, in comparison with KB-3.1 cells. To probe the role of efflux on carolacton’s cellular activity, we treated *E. coli* DSM-1116 (wt) and *E. coli* Δ*tolC* with a permeability enhancer and/or an efflux pump inhibitor (Table 2). When *E. coli* DSM-1116 was treated with a combination of carolacton and sub-inhibitory concentrations of polymyxin B nonapaptide (PMBN) to increase outer membrane permeability^33^, the MIC was still at the upmost range of the assay (at least 64 µg/mL). The same assay repeated with carolacton-susceptible *E. coli* Δ*tolC* showed a four-fold increase of carolacton activity. In contrast, when *E. coli* DSM-1116 was treated with carolacton and the efflux inhibitor phenylalanine–arginine β-naphthylamide (PAβN)^34^, the carolacton MIC decreased to 2 μg/mL. In addition, combining PMBN, carolacton and PAβN did not enhance the effect of carolacton further. These data support that carolacton can enter cells, but it is excreted by efflux proteins, which seems to be the main challenge to be addressed when attempting to increase the cellular activity of carolacton.

**Table 2:** MIC of carolacton on *Escherichia coli* determined in cation-adjusted Mueller–Hinton broth

	MIC (µg/mL)
*E. coli* (TolC-deficient)	0.13
*E. coli* (TolC-deficient) + PMBN^a^	≤0.03
*E. coli* DSM-1116 (WT)	>64
*E. coli* DSM-1116 (WT) + PMBN^a^	64 to >64
*E. coli* DSM-1116 (WT) + PAβN^b^	2^c^
*E. coli* DSM-1116 (WT) + PMBN^a^ + PAβN^b^	2^c^

^a^ + 3 µg/mL polymyxin B nonapaptide (PMBN) for permeabilization

^b^ + 20 µg/mL phenylalanine-arginine β-naphthylamide (PAβN; efflux inhibitor)

^c^ No full inhibition

## Discussion

We have identified the bifunctional enzyme FolD from the folate-dependent C1 metabolism as the target of carolacton. Through the analyses of carolacton-resistant *E. coli* Δ*tolC* mutants, biochemical experiments and the crystal structure of the FolD-carolacton complex, we were able to identify the key residues involved in carolacton binding (Y50, K54, and G261). All the residues mutated in carolacton-resistant *E. coli* Δ*tolC* isolates affected FolD’s DH activity only partially, but almost completely abolished its CYH activity. This reflects the fact that the DH activity is vital (no alternative pathway exists in *E. coli*), while the CYH reaction can also occur spontaneously. However, carolacton is effluxed in wt *E. coli* as evidenced by the activity on *E. coli* Δ*tolC* mutants, and the data show a strong enhancing effect of the efflux pump inhibitor PAβN.

Due to the high degree of sequence conservation between FolD and human mitochondrial hsMTHFD2, especially of the key carolacton-binding residues, we tested carolacton as a possible inhibitor of hsMTHFD2. This protein is overexpressed in many tumor cells^20^, and we observed strong inhibition and binding by carolacton in vitro. To see if carolacton could serve as a starting point for FolD/hsMTHFD2 inhibitors that display activity on whole cells, we tested the effect of carolacton on several cancer cell lines. Carolacton displayed moderate activity in these assays. Since carolacton is able to inhibit both reactions carried out by this enzyme and perturbs cofactor binding, we believe that carolacton, a non-substrate inhibitor, may be used as a tool compound to assess MTHFD2 as an anti-cancer target in the future.

## Methods

### Strains and plasmids

The *E. coli*Δ*tolC* strain used in carolacton-resistant mutant development is an antibiotic-sensitive *E. coli* strain with deficient outer membrane channel protein TolC. It was the same strain used in the previous carolacton studies^2, 4^. *E. coli* DH10B^35^ or DH5α (Invitrogen) strains were used for gene cloning and *E. coli* BL21 (DE3) (Novagen) was used for protein expression. *E. coli* DSM-1116 was ordered from German Collection of Microorganisms and Cell Cultures (DSMZ). The protein expression plasmid pHis-TEV^36^ was used for cloning and expression of all *folD* gene orthologues studied in this paper (Supplementary Table 9).

### Carolacton-resistant mutant development

First, the minimum inhibitory concentration (MIC) of carolacton on the *E. coli*Δ*tolC* strain was determined. We started to develop the resistant mutants on CASO agar (peptone from casein 15 g/L, peptone from soybean 5 g/L, NaCl 5 g/L and agar 15 g/L; pH 7.3) plates supplemented with 4× the MIC concentration of carolacton (0.5 μg/mL) as selection pressure. Different numbers of *E. coli*Δ*tolC* cells from 1 × 10^7^ to 10^9^ were spread on caso agar plates with carolacton. After 7 days, *E. coli*Δ*tolC* colonies, which were resistant to carolacton, started to form on the agar plates. The mutants picked were inoculated in liquid MHB medium (beef extract 2 g/L, peptone from casein 17.5 g/L and corn starch 1.5 g/L, pH 7.4) supplemented with carolacton. The genomic DNA of wt *E. coli*Δ*tolC* and five carolacton resistant mutants were extracted and the genomes sequenced.

### Whole-genome sequencing and variant calling

Genomic DNA of five independent mutant strains and one control *E. coli* Δ*tolC* strain was sequenced using Illumina Paired-End technology on the MiSeq instrument at the Helmholtz Centre for Infection Research (Braunschweig, Germany). Characteristics of the obtained raw-data sequencing reads are shown in Supplementary Table 1. Raw data were imported into the Geneious 9.1.3^37^ software package and trimmed of low-quality parts with an error probability threshold of 0.05. It was aligned against the *E. coli* Δ*tolC* reference sequence using the ‘Low sensitivity’ option of the Geneious ‘Map to reference…’ sequence aligner. This produced assembly files in which whole genome mean sequencing coverages varied in the range of 108–116. Assembly files were then converted to consensus sequences by the ‘Generate consensus sequence…’ option in the Geneious software and ‘Highest quality’ consensus calling option. Six resulting consensus sequences were aligned to each other and to the reference genome sequence by the ‘progressiveMauve’ algorithm of the MAUVE whole-genome sequence alignment tool^38^. The variant calling was done by comparing the consensus sequences of each mutant sample against the control *E. coli* Δ*tolC* WT consensus sequence. The complete list of mutations is shown in Supplementary Table 2.

### Gene cloning and protein purification

All *folD* gene orthologues studied in this paper were cloned into the pHis-TEV protein expression vector. The *folD* genes of *E. coli*Δ*tolC* and its carolacton-resistant mutants were amplified from the genomic DNA of the corresponding strains. The *folD* gene of *S. pneumoniae* was amplified from the genomic DNA of the TIGR4 strain^39^. The cDNAs encoding MTHFD1 and MTHFD2 were synthesized based on the available human genomic DNA sequences (gene ID 4522 and 10197, respectively; DNA synthesized by ATG:biosynthesis). All primers and restriction sites are listed in Supplementary Table 10. The resulting protein expression plasmids were verified by enzyme restriction digestion and DNA sequencing before being transformed into *E. coli* BL21 (DE3) for protein overexpression and purification. For optimal protein purification, a systematic buffer test including ten different buffers was conducted for each protein by using the KingFisher mL (ThermoFisher scientific) magnetic beads purification system. For large-scale protein purification, a single colony was picked into LB liquid medium containing 50 μg/mL kanamycin to make an overnight culture. The overnight culture was inoculated 1 to 100 into fresh LB medium supplemented with 50 μg/mL kanamycin and was grown at 37 °C until the optical density at 600 nm (OD_600_) reached 0.6. Then, the culture was transferred to 16 °C for half-an-hour to cool the culture before 0.1 mM IPTG was added to induce protein expression. The cells were harvested and lysed by sonication after 16 h shaking at 16 °C. The proteins were purified by immobilized metal ion affinity chromatography (IMAC) on a 5 mL Histrap HP column (GE healthcare). A HiPrep 26/10 desalting column (GE healthcare) was used to remove imidazole. When removal of the N-terminal 6x His-tag was required, TEV protease was added to the imidazole free protein solution and incubated at 4 °C overnight, followed by another hour at room temperature. The digestion mixture was loaded on a HisTrap HP column for a second time to bind the undigested protein, His-tag and TEV protease. The flow-through, which contained the protein, was collected, concentrated and loaded onto a gel filtration column HiLoad 16/600 Superdex 200 pg (GE healthcare) to further remove impurities and FolD aggregates. Finally, all purified proteins used in this study were checked by sodium dodecyl sulphate polyacrylamide gel electrophoresis (SDS-PAGE) and subjected to liquid chromatography–mass spectrometry (LC-MS) to check purity and assess protein molecular weights. All buffers for protein purification are listed in Supplementary Table 11.

### Enzyme assay conditions

(*6R,S*)*-*5,10-Methylene-5,6,7,8-tetrahydrofolic acid ((*6R,S*)*-*5,10-CH_2_-THF) calcium salt and (*6R,S*)*-*5,10-Methenyl-5,6,7,8-tetrahydrofolic acid ((*6R,S*)*-*5,10-CH=THF) chloride were purchased from Schircks Laboratories (Bauma, Switzerland) and used as substrates for FolD dehydrogenase and cyclohydrolase enzyme assays, respectively. Only the *R*-isomer, which accounts for 50% of (*6R,S*)*-*5,10-CH_2_-THF and (*6R,S*)*-*5,10-CH=THF, respectively, is used by the enzyme. (*6R,S*)*-*5,10-CH_2_-THF was dissolved in N_2_-spargeled basic buffer (50 mM Tris-HCl (pH 8.0), 100 mM β-mercaptoethanol) as described by Varshney et al.^25^. (*6R,S*)*-*5,10-CH=THF was dissolved in DMSO as a 100 mM stock solution.

Because FolD is a bifunctional enzyme, dehydrogenase and cyclohydrolase activities were determined for FolD enzyme kinetics, respectively. The dehydrogenase activity of FolD was assayed for its substrate 5,10-CH_2_-THF and cofactor NADP^+^ (in the case of MTHFD2, NAD^+^ was used as the cofactor) based on monitoring the formation of 5,10-CH=THF, while the cyclohydrolase activity of FolD was determined for its substrate 5,10-CH=THF by monitoring the hydrolysis of 5,10-CH_2_-THF. The enzyme kinetics for the dehydrogenase activity of all FolDs except hsMTHFD1_DC were determined at 30 °C in 50 mM Tris-HCl (pH 7.5), 30 mM β-mercaptoethanol. The hsMTHFD1_DC dehydrogenase activity was measured in 25 mM MOPS (pH 7.3), 30 mM β-mercaptoethanol at 30 °C. To measure the enzyme kinetic constants for 5,10-CH_2_-THF, for example in the case of ecFolD, the NADP concentration was fixed at 1 mM and the concentration of 5,10-CH_2_-THF varied from 5 to 1500 μM (*R-*isomer). Similarly, to measure the kinetic constants of ecFolD for NADP^+^, the concentration of 5,10-CH_2_-THF (*R*-isomer) was fixed at 1 mM and the concentration of NADP^+^ varied from 10 to 1500 μM. For different FolDs, the varied concentration range of substrates could be different. The 50 μL reactions of dehydrogenase assays were initiated by adding appropriate amounts enzyme (4 nM ecFolD, 4 nM spFolD, 15 nM hsMTHFD1_DC, 20 nM hsMTHFD2, 20 nM G8S, 20 nM Q98H, 10 nM K54N and 200 nM ΔK54R55 in the corresponding assays) and terminated with 50 μL 1 M HCl after 2 min incubation at 30 °C. To monitor the formation of 5,10-CH=THF, the absorbance of the reaction mixture was monitored at 350 nm in micro UV cuvettes (BRAND, Essex, Connecticut, USA). The concentration of 5,10-CH=THF produced in the reaction was determined using an extinction co-efficient of 0.0249 μM^−1^ cm^−1^. Cyclohydrolase activity for each FolD was assayed in the same buffer as dehydrogenase activity at 30 °C but for 30 s. The concentration of FolD used in each assay was 5 nM of ecFolD, spFolD and hsMTHFD1_DC, respectively, and 2 nM of hsMTHFD2. The consumption of 5,10-CH=THF was measured by recording the absorbance of the reactions at 355 nm using the TECAN Infinite 200 PRO equipped with a monochromator. To measure the enzyme kinetic constants, different concentrations of 5,10-CH=THF (varied from 0.5 μM to 100 μM) were used in 100 μL reaction mixtures. The inhibition constants (*K*_*i*_) of carolacton on FolD were determined by measuring the apparent *K*_m_ when adding certain amounts of carolacton to the enzyme reactions. For ecFolD, 10 nM carolacton was added in the dehydrogenase assay and 50 nM carolacton was used in the cyclohydrolase assay. For spFolD, 25 nM carolacton was added in both dehydrogenase assay and cyclohydrolase assay. For hsMTHFD1_DC, 30 nM carolacton was added in the dehydrogenase assay and 10 nM in the cyclohydrolase assay. For hsMTHFD2, 20 nM carolacton was used to determine the apparent *K*_m_ for 5,10-CH_2_-THF and 5,10-CH_2_-THF, while 10 nM carolacton was used to determine the apparent *K*_m_ for NADP. To compare the inhibition effects of carolacton between the different FolDs, the half maximal inhibitory concentration (IC_50_) of carolacton on the same amount of enzyme was determined for FolDs involved in this study. The enzyme concentration used to determine dehydrogenase activity IC_50_ was 10 nM, and the enzyme used for cyclohydrolase IC_50_ measurements was 5 nM. All enzyme kinetics measurements were performed as independent triplicates. Data processing and fitting of curves was done using OriginPro 2016 (OriginLab, Northampton, Massachusetts, US). Analysis of variance (ANOVA) in OriginPro 2016 software gave statistical test reports after fitting curves.

### Surface plasmon resonance assay

All surface plasmon resonance (SPR) experiments described in this study were performed on a Biacore X100 system. Different FolDs with or without His-tag were coupled on CM5 sensor chips (GE Healthcare) by the amine coupling method using a kit from GE Healthcare Life Sciences (Freiburg, Germany). A pH scouting process was performed before protein immobilization to test the most appropriate buffer and the protein concentration for protein coupling. Finally, 20 μg/mL of ecFolD in 10 mM maleate buffer (pH 6.8), 30 μg/mL of ecFolD Q98H in 10 mM maleate buffer (pH 6.3), 40 μg/mL of ecFolD K54N in 10 mM maleate buffer (pH 5.8), 20 μg/mL of spFolD in 10 mM maleate buffer (pH 6.0) and 15 μg/mL of MTHFD2 (His-tag cleaved) in 10 mM maleate buffer (pH 6.8) were used for coupling. In the protein immobilization procedure, the contact time was calculated based on the pH scouting results to achieve ~3000–5000 relative resonance units (RU). The buffer used in the SPR assays was 1× HBS-P buffer (10 mM HEPES pH 7.4, 150 mM NaCl, 0.05% Tween 20). The 1 mM stock solution of carolacton was prepared in 1× HBS-P buffer and diluted to different concentration ranges by two-fold serial dilution. In the SPR assay, the association time was 180 s and the dissociation time was 600 s. Data analysis was performed using the Biacore evaluation software. The SPR curves were exported to be re-plotted in OriginPro 2016 (OriginLab, Northampton, Massachusetts, US).

### Antimicrobial screening

The determination of MICs was performed as described elsewhere^40^. In brief, *E. coli* strains were treated with carolacton in serial dilution in either the presence or absence of sub-inhibitory concentrations of polymyxin B nonapeptide (PMBN; 3 µg/mL) and/or phenylalanine–arginine β-napthylamide (PAβN; 20 µg/mL) for 16 h at 37 °C in Mueller–Hinton broth. The MICs were assessed by visual inspection of the plates.

### Cytotoxicity screening

Half maximal effective concentrations (EC_50_) on human cancer cell lines were determined as described elsewhere^41^. Human cancer cell lines were maintained as recommended by the depositor (German Collection of Microorganisms and Cell Cultures, DSMZ) and were treated for 5 d with carolacton in serial dilution. The viability of adherent cell types, HCT-116 (ACC 581), KB-3.1 (ACC-158) and KB-V.1 (ACC-149) was assessed by the addition of MTT tetrazolium salt and absorbance measurement at 570 nm. Histiocytic lymphoma U-937 cells (ACC-5) were maintained in RPMI 1640 medium and transferred into folate-free medium prior to the assay. Cell viability was determined by measuring alamar blue fluorescence emission at 590 nm. The values were referenced and EC_50_ values were determined by sigmoidal curve fitting. The cell lines KB-3.1 and KB-V.1 are originally derived from HeLa, whereas the latter is an MDR subclone of KB-3.1. They were used to directly compare the activity of carolacton on an *mdr1* overexpressing and a sensitive cell line. The effect of folate depletion was assessed using the U-937 cell line as these lymphoma cells are described to express high levels of MTHFD2, however, carolacton was virtually inactive under normal culture conditions.

### Reductive methylation of surface lysine residues

To protect active site lysine K54, a six-fold molar excess of carolacton was added to the protein and the complex incubated on ice for 16 h before the reaction. Surface lysine residues were then methylated using the JBS Methylation Kit (Jena Bioscience) according to the manufacturer’s instructions and Supplementary Information. Methylated ecFolD (ecFolD^Meth^) and ecFolD^Meth^Q98H were applied to a Superdex 200 gel filtration column (GE Healthcare) pre-equilibrated with gel filtration buffer (150 mM NaCl, 10 mM HEPES (pH 7.4) and 1 mM TCEP), and then concentrated to 5 mg/mL.

### X-ray crystallography

Crystals of ecFolD^Meth^ were obtained at 18 °C by using the sitting drop vapour diffusion method. Protein (300 nL) at 5 mg/mL was added to 150 nL reservoir solution: 0.2 M sodium acetate, 0.1 M sodium cacodylate pH 6.5 and 30% (w/v) PEG 8000. To solve the ecFolD-carolacton complex structure, ecFolD^Meth^ crystals were soaked overnight in the presence 2 mM carolacton. Optimized crystals of apo-ecFolD^Meth^Q98H were grown under conditions of 0.2 ammonium sulphate, Bis-Tris pH 6.0 and 25% PEG 3350. Diffraction data for all proteins were collected from single crystals at 100 K. Data for ecFolD^Meth^ were obtained at Beamline X06DA at a wavelength of 1 Å (Swiss Light Source), while data for the ecFolD^Meth^–carolacton complex and ecFold^Meth^Q98H crystals were collected at beamline ID30-A3 (ESRF) at a wavelength of 0.967 Å. Data were processed using Xia2^42^ or XDS^43^ and the structures solved using PHASER^44^ Molecular replacement with ecFolD (PDB ID 1DIA) as a search model. The models were manually rebuilt in COOT^45^ and refined using PHENIX^46^ and Refmac5^47^ (Supplementary Table 12). The structures were validated using *MolProbity*, and all images presented were created using PyMOL^48^. Interaction diagrams were created using Ligplot^49^.

### Data availability

Atomic coordinates and structure factors are deposited in the RCSB Protein Data Bank with accession codes 5O28 (ecFolD apo), 5O22 (ecFolD carolacton complex) and 5O2A (ecFolDQ98H). Other relevant data supporting the findings of this study are available in this published article and its Supplementary Information files or from the corresponding authors upon request.

## Electronic supplementary material

Supplementary Information
