# Supplementary material for: The natural product carolacton inhibits folate-dependent C1 metabolism by targeting FolD/MTHFD
Source: Nat Commun. 2017 Nov 16;8:1529. doi: 10.1038/s41467-017-01671-5 (PMC5688156; doi:10.1038/s41467-017-01671-5)
Supplement: Supplementary file 1 — Supplementary Information [file 41467_2017_1671_MOESM1_ESM.pdf]

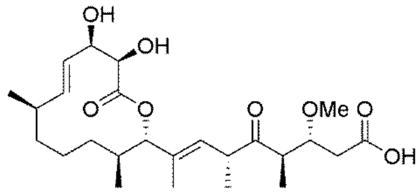

Carolacton

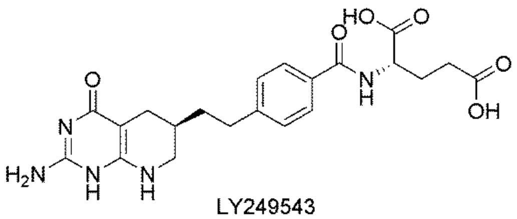

LY249543

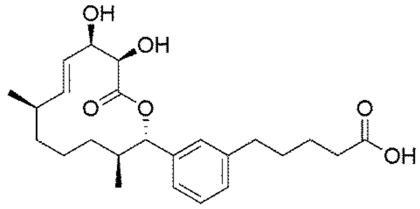

Carylacton

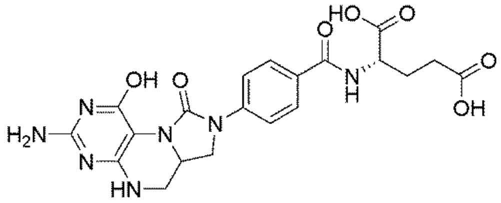

LY345899

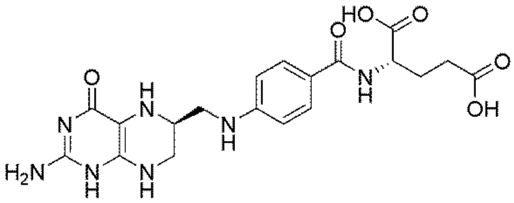

Tetrahydrofolate

Supplementary Figure 1. Chemical structures of compounds mentioned in this paper.

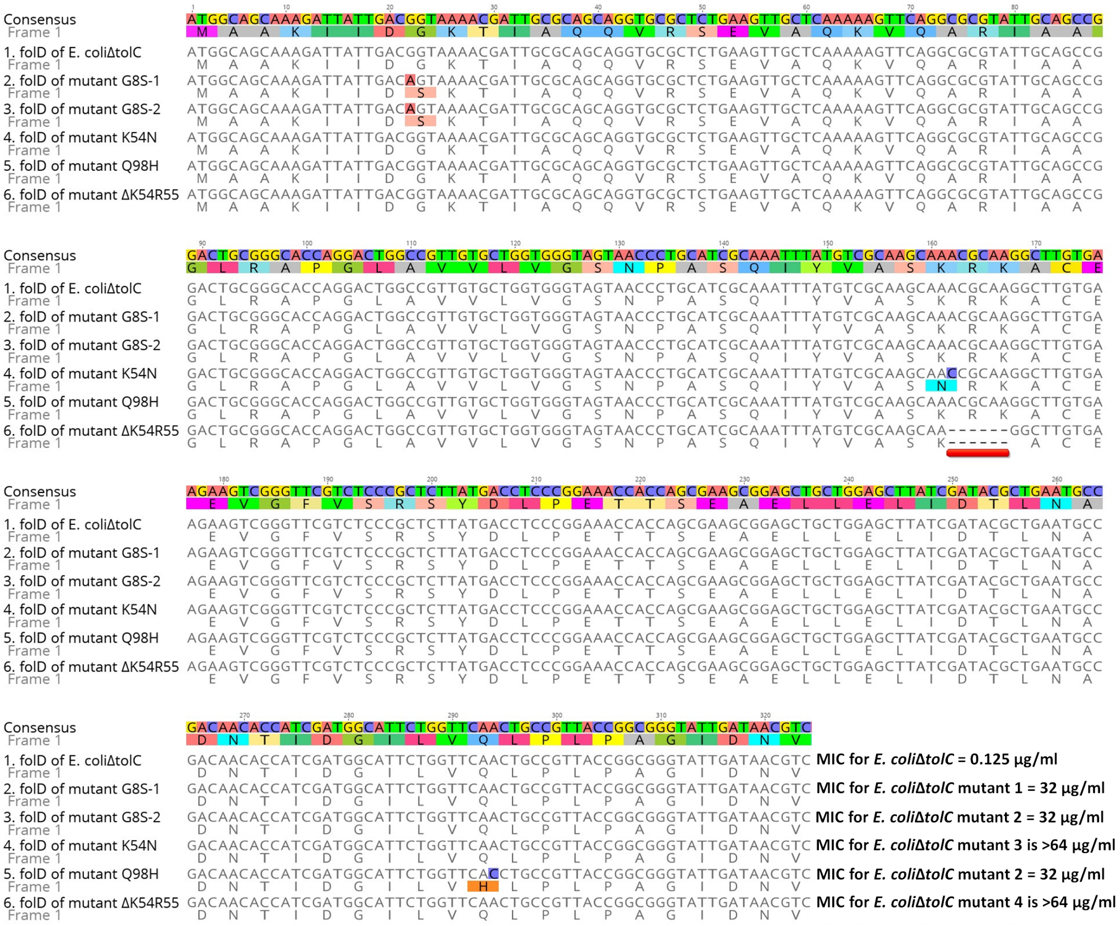

Supplementary Figure 2. Partial alignment of *folD* genes and MIC of *E. coli*Δ*tolC* wt and four carolacton-resistant mutants. All point mutations and base deletions were highlighted in different colours. The *folD* gene of mutant ΔK54R55 has a 6 bp deletion affecting the codons for Lysine 54, Arginine 55 and Lysine 56. The MIC values of carolacton against *E. coli*Δ*tolC* wt strain and five carolacton-resistant mutants are shown at the end of alignments.

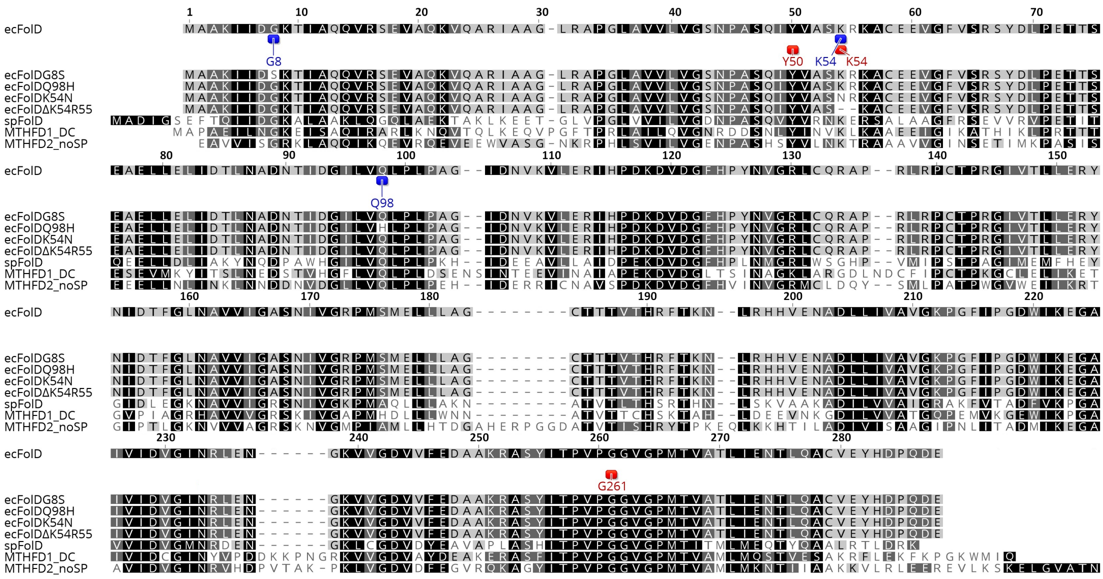

Supplementary Figure 3. Alignment of FolD enzymes studied in this paper. The residues indicated by blue squares are residues mutated in FolD proteins from carolacton-resistant mutants. The residues indicated by red squares are important for carolacton binding. The numbering of residues is according to ecFolD.

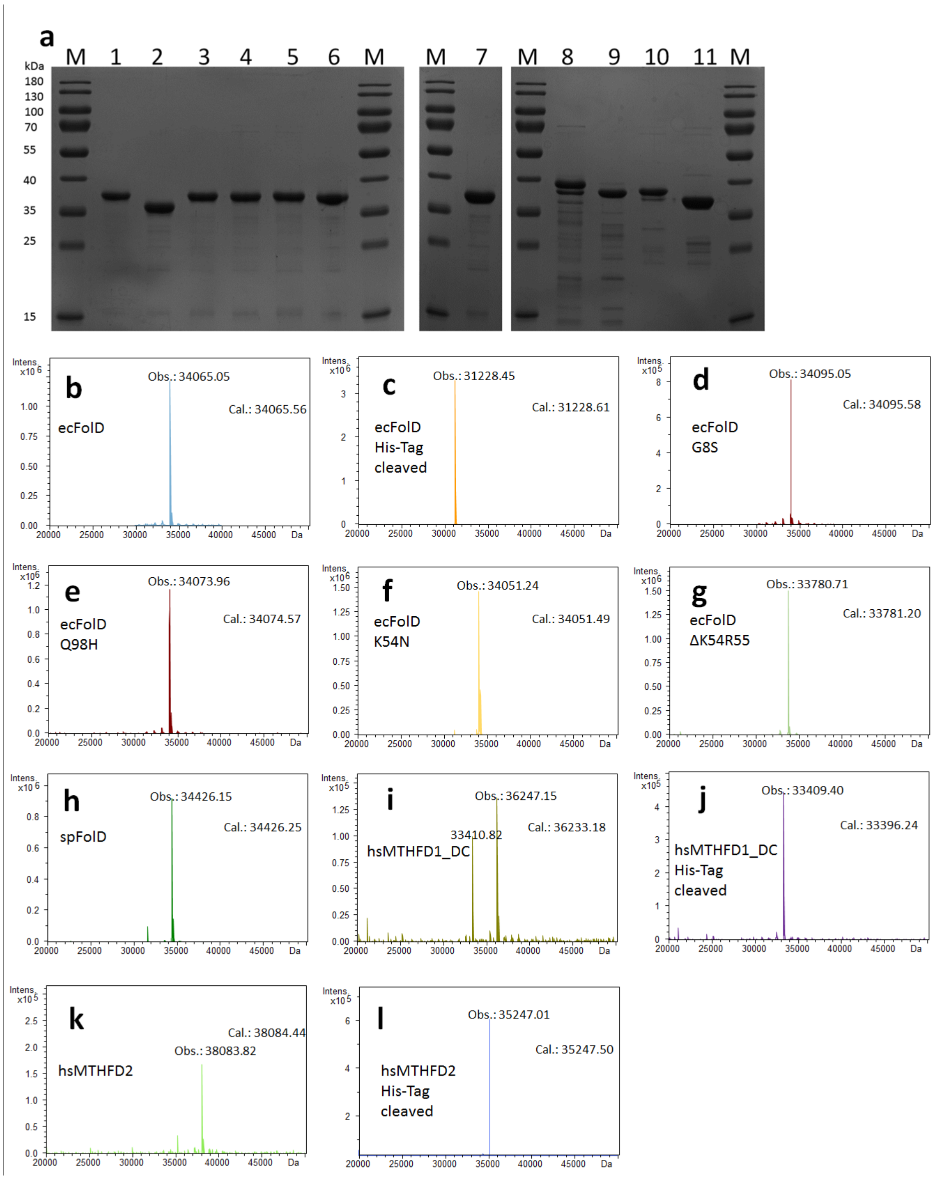

Supplementary Figure 4. All purified FolD proteins analyzed by SDS-PAGE and LC-MS. (**a**) The SDS-PAGE gel of all purified FolDs. M: PageRuler prestained protein ladder; 1: His-Tag fusion FolD of *E. coli*Δ*tolC*; 2: FolD of *E. coli*Δ*tolC* (His-tag cleaved off) 3: His-Tag fusion FolD of *E. coli*Δ*tolC* G8S; 4: His-Tag fusion FolD of *E. coli*Δ*tolC* Q98H; 5: His-Tag fusion FolD of *E. coli E. coli*Δ*tolC* K54N; 6: His-Tag fusion FolD of *E. coli*Δ*tolC* ΔK54R55; 7: His-Tag fusion FolD of *S. pneumoniae* TIGR4; 8: His-Tag fusion hsMTHFD1_DC; 9: His-Tag cleaved hsMTHFD1_DC; 10: His-Tag fusion hsMTHFD2; 11: His-Tag cleaved hsMTHFD2. (**b-l**) Deconvoluted mass spectra of all FolD proteins. Cal. stands for calculated average neutral mass according to the protein sequence; Obs. indicates the average neutral mass observed by LC-MS measurement. The mass of hsMTHFD1_DC is 14 Da heavier than its calculated mass, which is possibly the result of methylation on one residue.

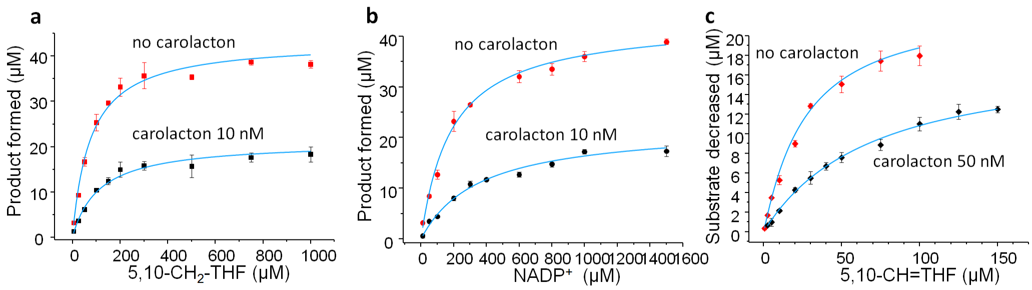

Supplementary Figure 5. Enzyme kinetics of ecFolD. Data are presented as means ± s.e.m of 3 independent replicates. Enzyme specific activity values were calculated based on the V_{max} values obtained via Michaelis-Menten or Hill fitting. The one-way ANOVA test was used for statistical analysis, P<0.01. (**a**) The determination of 5,10-CH_2-THF K_M in the presence of 1 mM $NADP^+$ and 5,10-CH_2-THF apparent K_M in the presence of 1 mM $NADP^+$ and 10 nM carolacton. (**b**) The determination of $NADP^+$ K_M in the presence of 1 mM 5,10-CH_2-THF and $NADP^+$ apparent K_M in the presence of 1 mM 5,10-CH_2-THF and 10 nM carolacton. (**c**) The determination of 5,10-CH=THF K_M and the determination of 5,10-CH=THF apparent K_M in the presence of 50 nM carolacton.

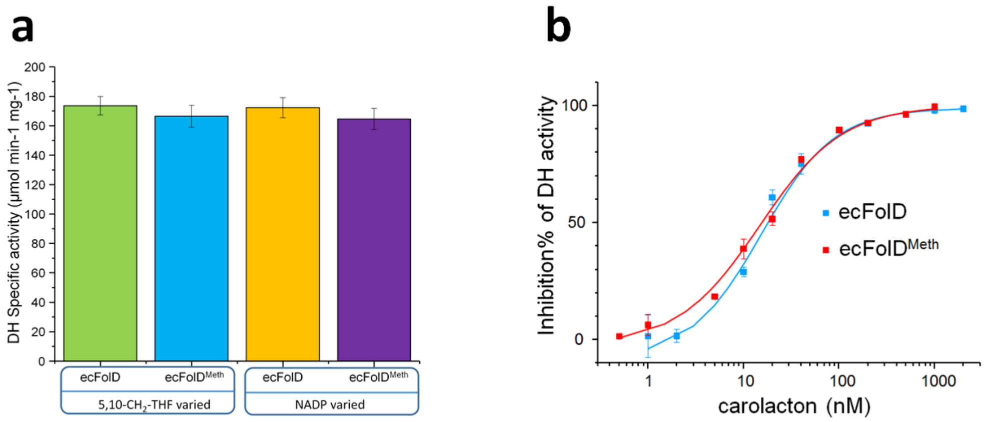

Supplementary Figure 6. The comparison between ecFolD and ecFolD^{Meth} (The lysine methylation processed ecFolD). (a) The column plot for the comparison of DH activity of ecFolD and ecFolD^{Meth}, the specific activity calculated based on varied 5,10-CH_2-THF and NADP$^+$ both showed. Data are presented as means ± s.e.m of 3 independent replicates. DH specific activity values were calculated based on the V_{max} values obtained via Michaelis-Menten fitting. The one-way ANOVA test was used for statistical analysis, P<0.01. (b) The determination of IC_{50} for carolacton inhibition against ecFolD and ecFolD^{Meth}. Data are presented as means ± s.e.m of 3 independent replicates. IC_{50} values were obtained via logistic dose–response fitting. The one-way ANOVA test was used for statistical analysis, $P < 0.01$.

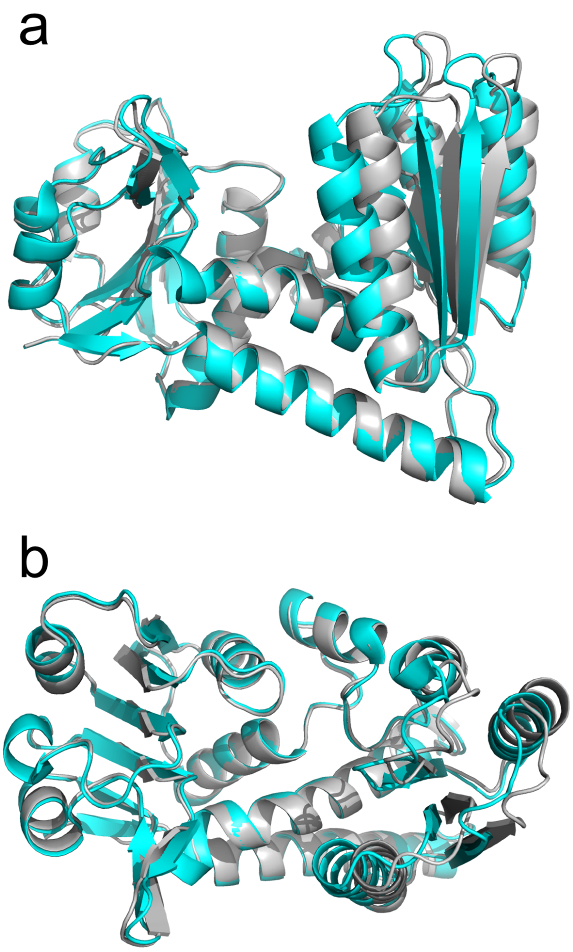

Supplementary Figure 7. Superposition of ecFolD crystallized in this study (gray) with the published structure (PDB ID 1B0A, cyan). (**a**) Side view. (**b**) Structures rotated by 90 ° around the horizontal axis towards the viewer.

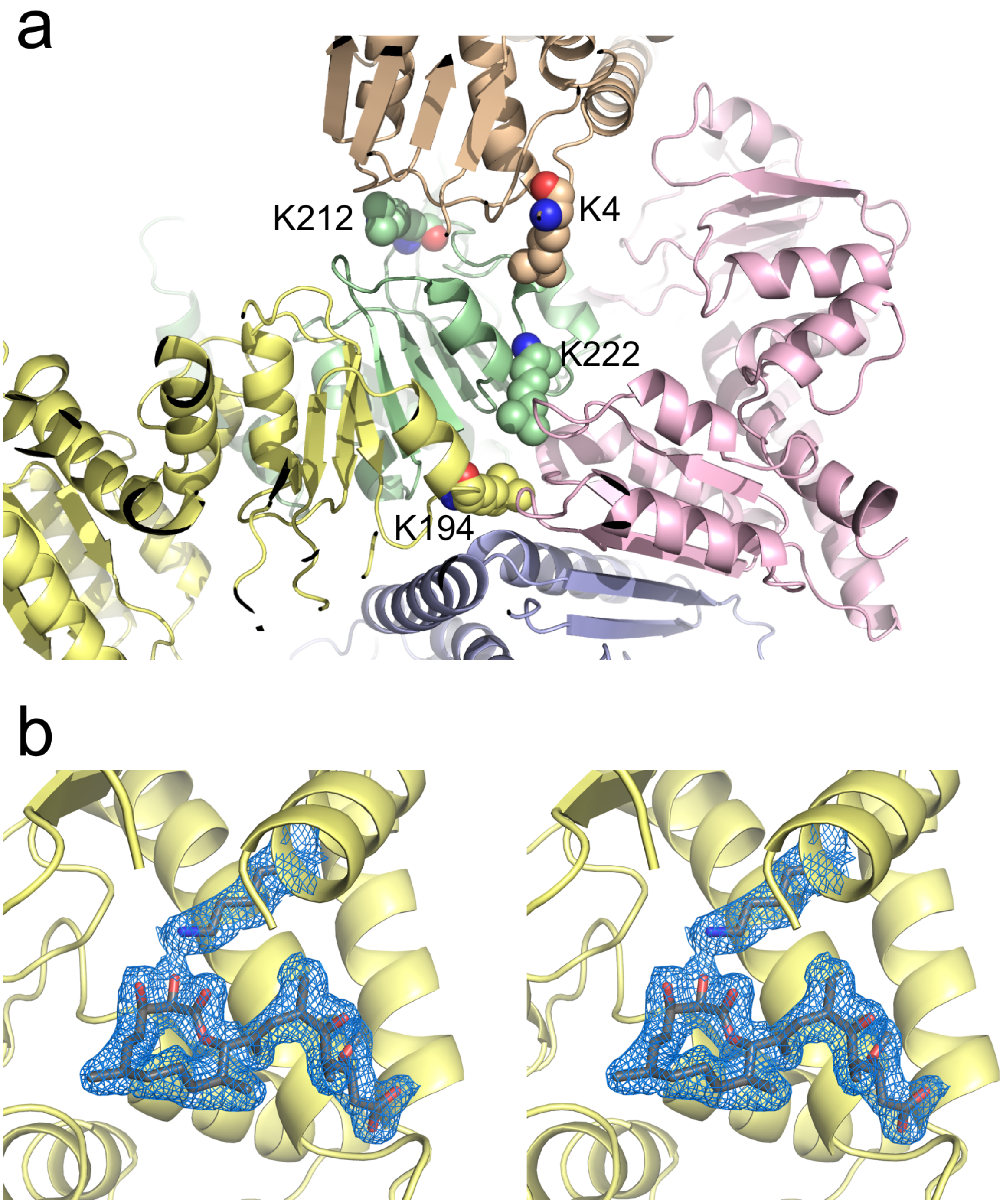

Supplementary Figure 8. (a) Lysine methylation is essential for the ecFolD crystal structures reported in this study. ecFolD protomers are shown as cartoon representations in individual colors. Dimethylated lysine residues involved in crystal contacts are shown as spheres with their color corresponding to the protomer they belong to. **(b)** Stereo view of the 2Fo-Fc electron density map for carolacton and K54. ecFolD is shown as a yellow cartoon, carolacton and K54 as sticks and the electron density as a blue isomesh.

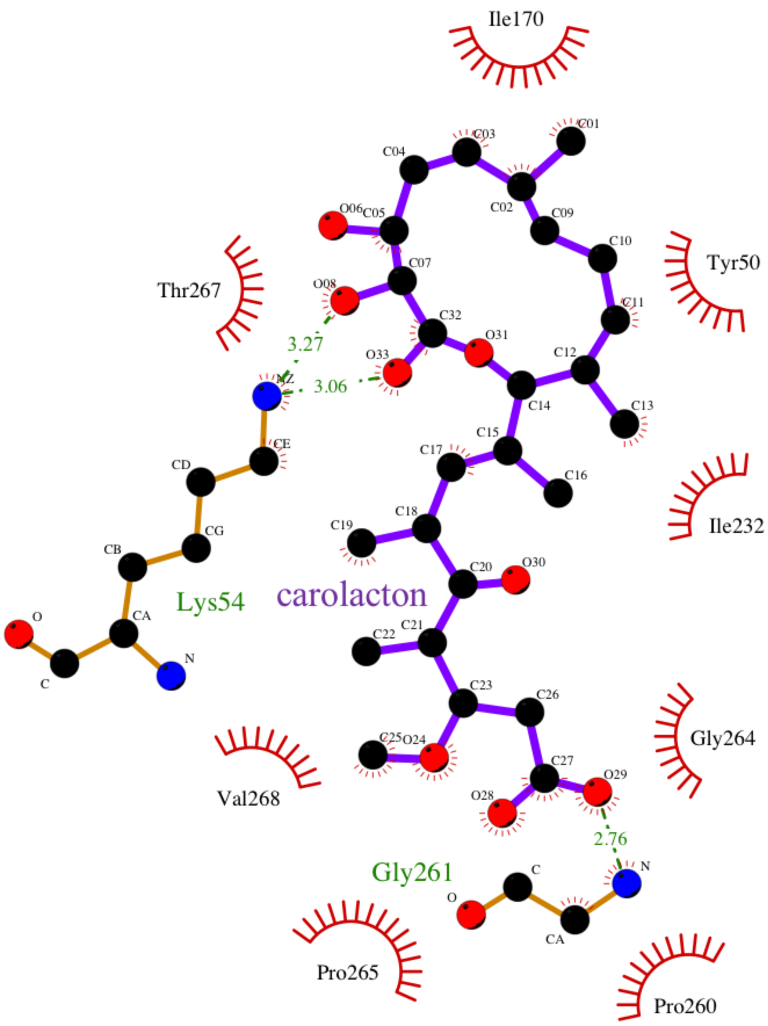

Supplementary Figure 9. Ligplot diagram showing the detailed interactions of carolacton with ecFolD.

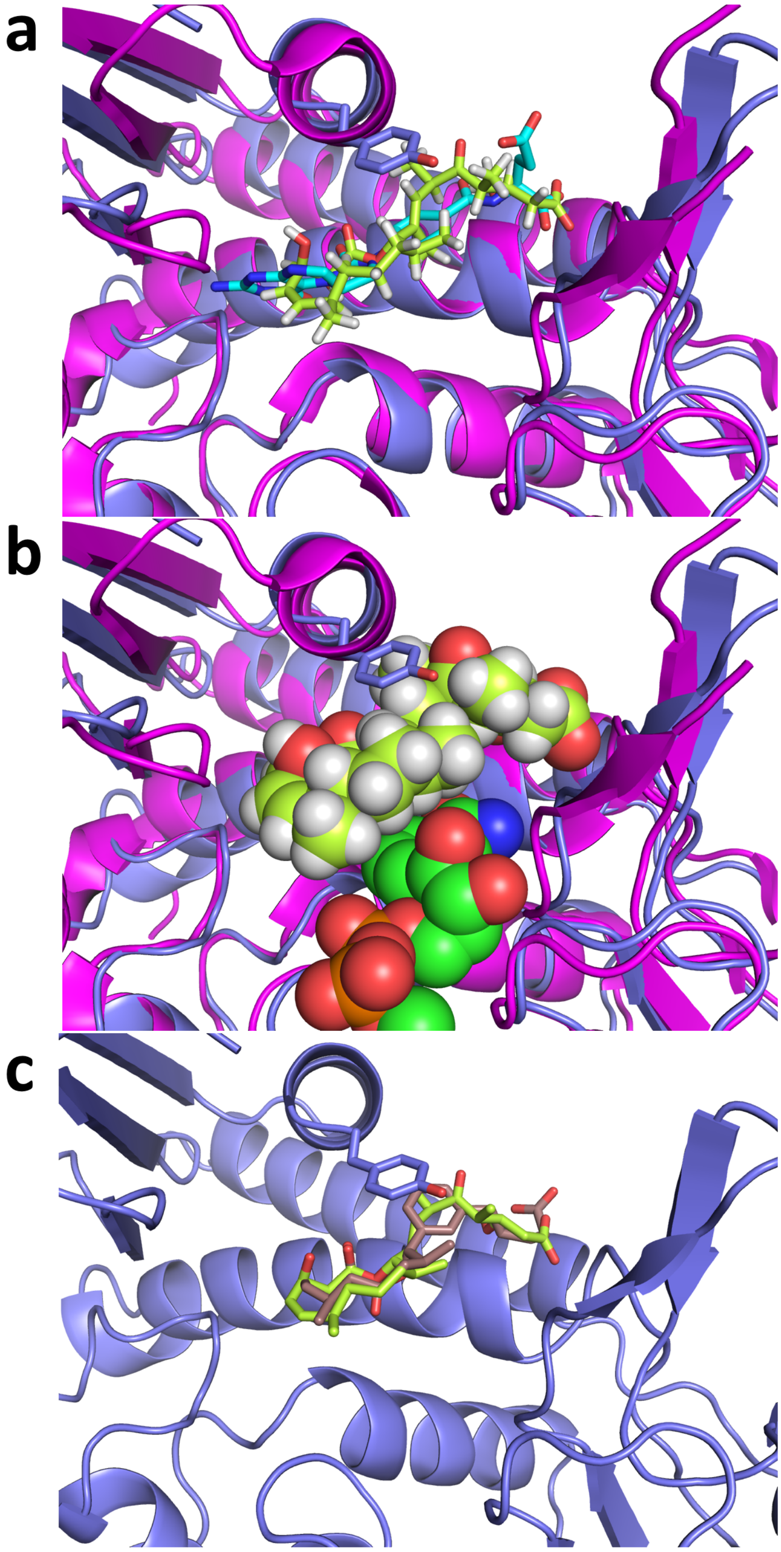

Supplementary Figure 10. Superposition of ecFolD (blue) with hsMTHFD2 (PDB ID 1DIA, magenta). (**a**) Carolacton (lime) clashes with the bound substrate analog L345899 (cyan). (**b**) carolacton clashes with co-factor NAD^+ (green). (**c**) New carolacton analog "carylacton" can engage with Y50 but has a tail which is too long to hydrogen-bond with G261.

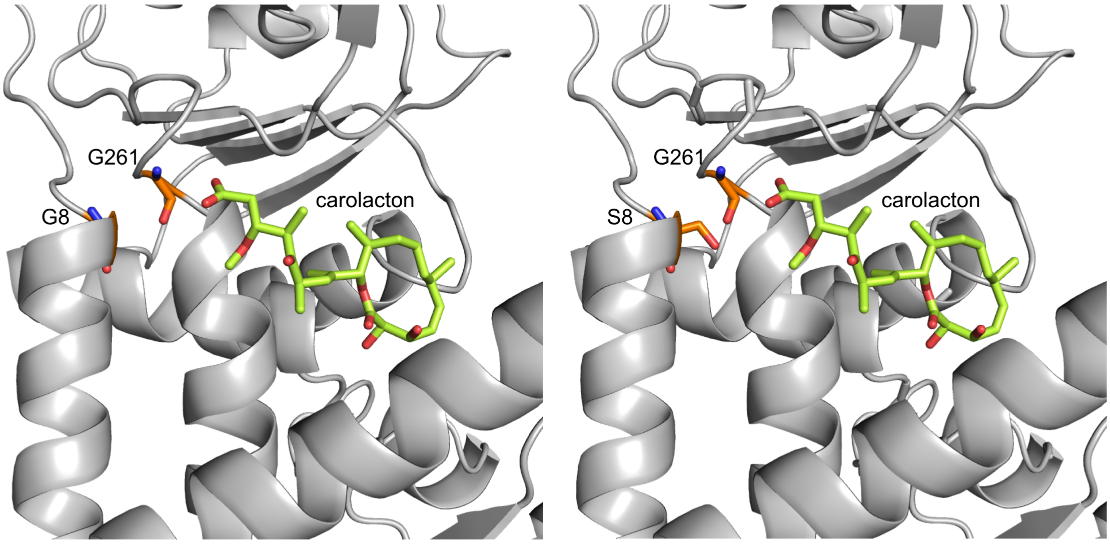

Supplementary Figure 11. Effect of mutation G8S on carolacton binding of ecFolD. Left: In wt ecFolD (gray) G8 is hydrogen-bonded to G261 (both orange), which in turn forms hydrogen bonds with carolacton (lime). **Right**: In the mutant G8S, the side-chain of the serine clashes with G261 in all conformations. Therefore, the loop containing G261 needs to move to accommodate S8, which results in a clash with carolacton.

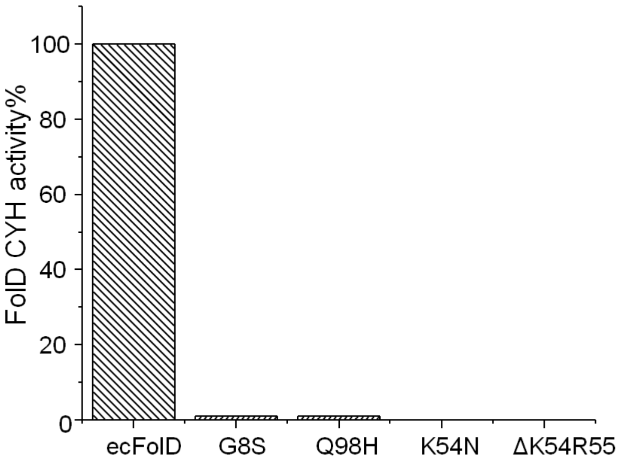

Supplementary Figure 12. Comparison of ecFolD CYH activity between wt and the carolacton-resistant mutants.

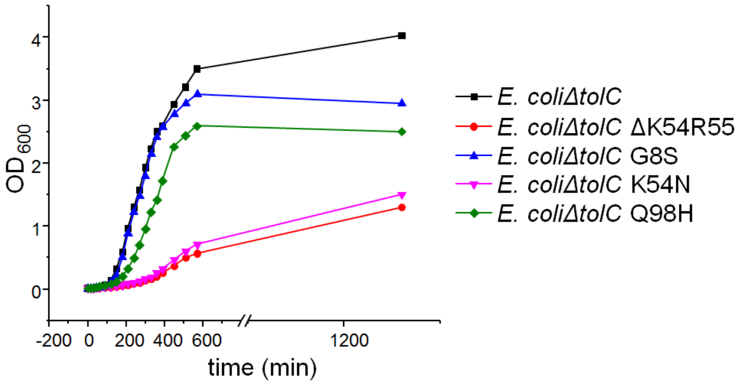

Supplementary Figure 13. Growth curves of *E.coli* Δ*tolC* and its carolacton-resistant mutant strains.

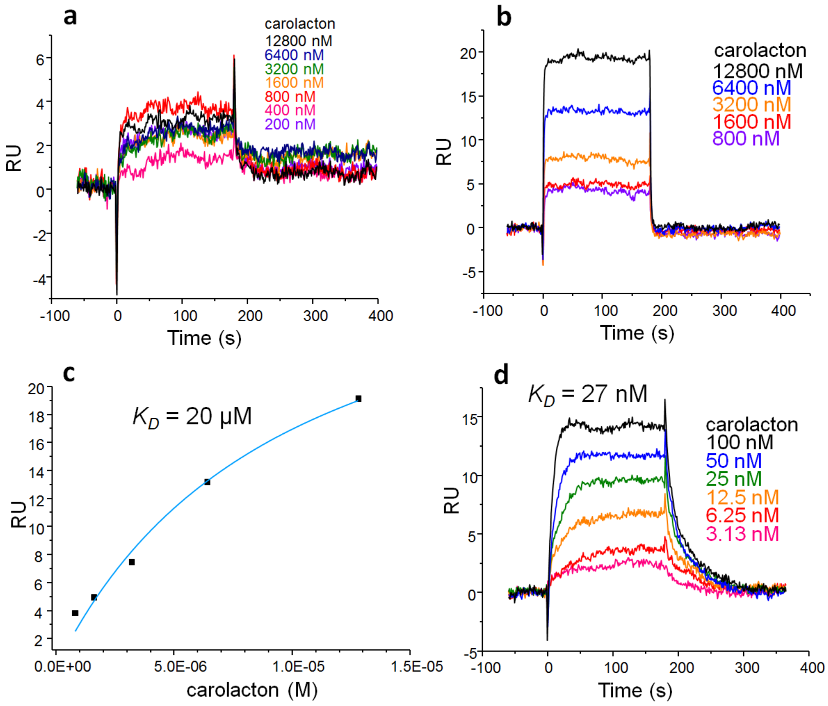

Supplementary Figure 14. SPR assays of spFolD and ecFolD mutants with carolacton. (**a**) SPR analysis shows carolacton does not bind to ecFolDK54N. (**b**) SPR analysis shows carolacton has weak binding to ecFolDQ98H, the dissociation of carolacton is much faster than wt ecFolD, but the K_D cannot be determined uniquely by SPR kinetics calculations. (**c**) The affinity calculation based on SPR analysis of carolacton binding to ecFolD Q98H gives a K_D which is 20 µM. (**d**) SPR analysis of carolacton binding to spFolD.

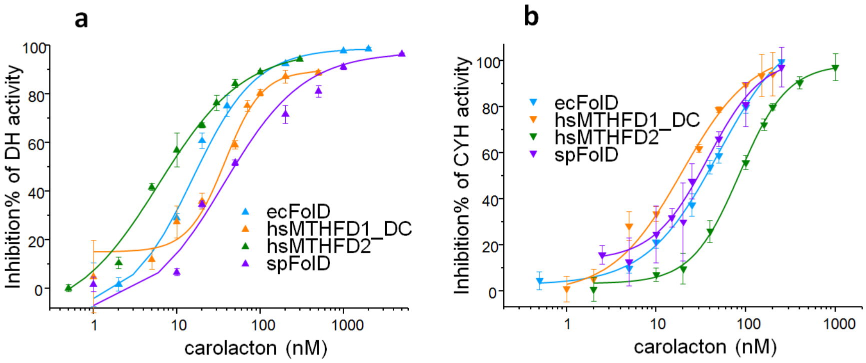

Supplementary Figure 15. The determination of IC_{50} for carolacton inhibition against all wt FolDs in this study. Data are presented as means ± s.e.m of 3 independent replicates. IC_{50} values were obtained via logistic dose–response fitting. The one-way ANOVA test was used for statistical analysis, $P < 0.01$. (**a**) IC_{50} determination for carolacton against the dehydrogenase activity of FolD from different organisms. (**b**) IC_{50} determination for carolacton against the cyclohydrolase activity of FolD from different organisms.

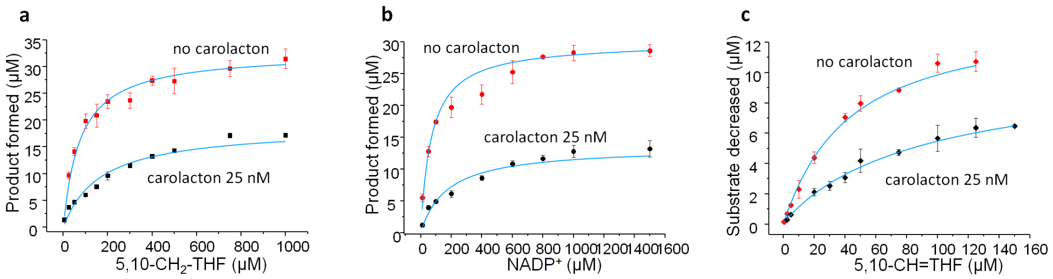

Supplementary Figure 16. Enzyme kinetics of spFolD. Data are presented as means ± s.e.m of 3 independent replicates. Enzyme specific activity values were calculated based on the V_{max} values obtained via Michaelis-Menten fitting. The one-way ANOVA test was used for statistical analysis, $P<0.01$. (**a**) The determination of 5,10-CH_2-THF K_M in the presence of 1 mM NADP and 5,10-CH_2-THF apparent K_M in the presence of 1 mM NADP and 25 nM carolacton. (**b**) The determination of NADP K_M in the presence of 1 mM 5,10-CH_2-THF and NADP apparent K_M in the presence of 1 mM 5,10-CH_2-THF and 25 nM carolacton. (**c**) The determination of 5,10-CH=THF K_M and the determination of 5,10-CH=THF apparent K_M in the presence of 25 nM carolacton.

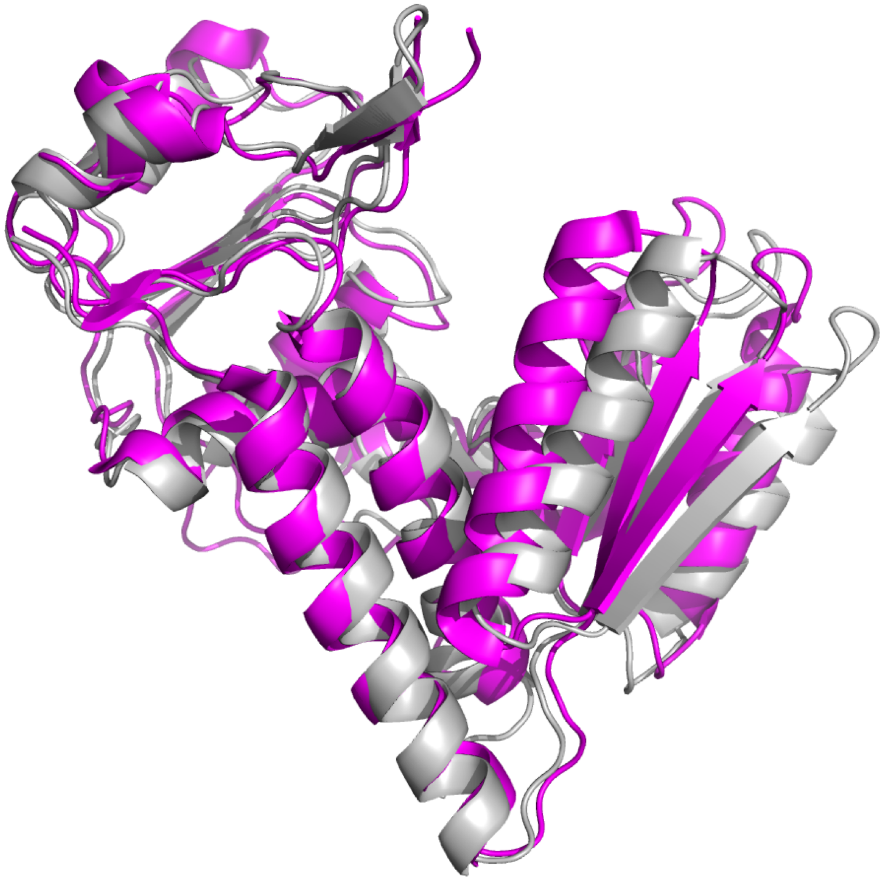

Supplementary Figure 17. Superposition of ecFolD (gray) with human mitochondrial hsMTHFD2 (PDB ID 5TC4, magenta).

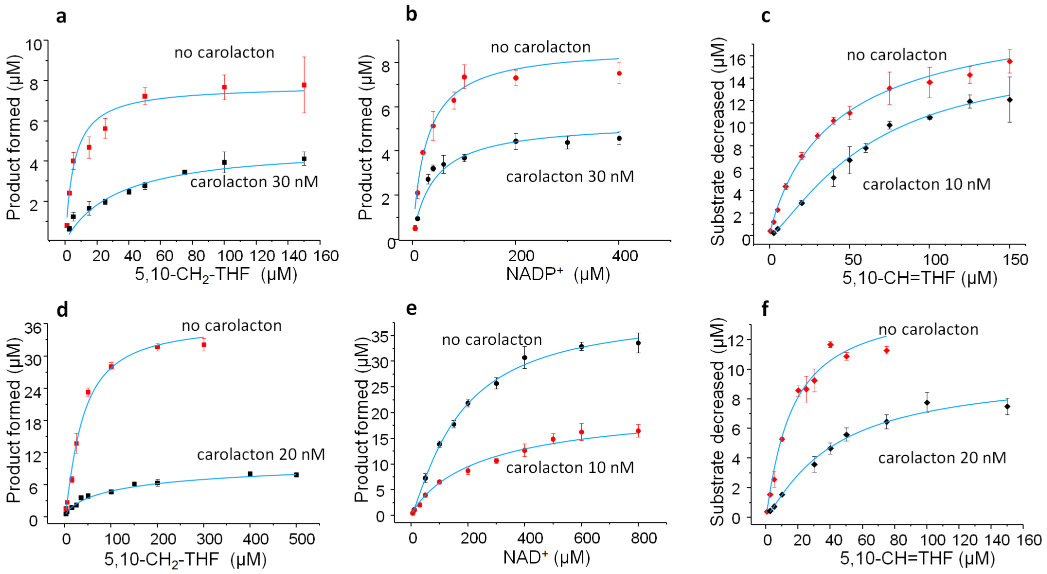

Supplementary Figure 18. The enzyme kinetics measurement of hsMTHFD1_DC and hsMTHFD2. Data are presented as means ± s.e.m of 3 independent replicates. Enzyme specific activity values were calculated based on the V_{max} values obtained via Michaelis-Menten or Hill fitting. The one-way ANOVA test was used for statistical analysis, $P<0.01$. (**a**) The determination of 5,10-CH_2-THF K_M for hsMTHFD1_DC in the presence of 0.4 mM NADP and 5,10-CH_2-THF apparent K_M for hsMTHFD1_DC in the presence of 0.4 mM NADP and 30 nM carolacton. (**b**) The determination of NADP K_M for hsMTHFD1_DC in the presence of 0.4 mM 5,10-CH_2-THF and NADP apparent K_M for hsMTHFD1_DC in the presence of 0.4 mM 5,10-CH_2-THF and 25 nM carolacton. (**c**) The determination of 5,10-CH=THF K_M for hsMTHFD1_DC and the determination of 5,10-CH=THF apparent K_M for hsMTHFD1_DC in the presence of 25 nM carolacton. (**d**) The determination of 5,10-CH_2-THF K_M for hsMTHFD2 in the presence of 0.6 mM NADP and 5,10-CH_2-THF apparent K_M for hsMTHFD2 in the presence of 0.6 mM NADP and 20 nM carolacton. (**e**) The determination of NADP K_M for hsMTHFD2 in the presence of 0.6 mM 5,10-CH_2-THF and NADP apparent K_M for hsMTHFD2 in the presence of 0.6 mM 5,10-CH_2-THF and 10 nM carolacton. (**f**) The determination of 5,10-CH=THF K_M for hsMTHFD2 and the determination of 5,10-CH=THF apparent K_M for hsMTHFD2 in the presence of 20 nM carolacton.

Supplementary Table 1. Characteristics of data obtained during whole genome sequencing and analysis.

**Sample**	Mutant 1	Mutant 2	Mutant 3	Mutant 4	Mutant 5	WT
**Technology**	MiSeq, Illumina, Paired-End					
**Number of reads**	2,426,032	2,423,998	2,376,598	2,513,800	2,314,528	2,372,346
**Read Length, bp**	251					
**Genome size, bp**	4,641,652 (expected)					
**Coverage (theoretical)**	131	131	128	135	125	128
**Coverage (post-assembly)**	108	108	108	116	107	109

Supplementary Table 2. List of mutations found in carolacton-resistant mutants (CaroM1-CaroM5) when compared to the control sample (WT).

Gene	Mutation	CaroM1	CaroM2	CaroM3	CaroM4	CaroM5	WT
bifunctional 5,10-methylenetetetrahydrofolate dehydrogenase/ 5,10-methenyltetrahydrofolate cyclohydrolase (FolD)	G8S		+	+			
	K54N				+		
	Q98H					+	
	K54_K56delinsK	+					

Supplementary Table 3. The specific activity of dehydrogenase and cyclohydrolase of bacterial FolD reported in current and previous studies.

Bacteria	FolD	DH activity (specific activity µmol min^{-1} mg^{-1})	CYH activity (specific activity µmol min^{-1} mg^{-1})	Reference
E. coli	ecFolD	173.57 ± 6.32	298.81 ± 37.87	This study
Acinetobacter baumannii	abFolD	161.4 ± 5.7	350.2 ± 4.4	Eadsforth et al.[1]
E. coli	ecFolD	200	33	D'Ari et al.[2]
E. coli	ecFolD	31.2	6.12	Dev et al.[3]
E. coli	ecFolD	19	39	Sah et al.[4]
Peptostreptococcus productus	ppFolD	627	ND	Wohlfarth et al.[5]
Clostridium formicoaceticum	cfFolD	ND	469	Clark et al.[6]

Supplementary Table 4. The inhibition constants of carolacton against all wt FolD in this study.

enzyme	carolacton inhibition on DH activity		carolacton inhibition on CYH activity
	K_i (nM) (5, 10-CH_2-THF)	K_i (nM) ($NADP^+$)[a]	K_i (nM) (5, 10-CH=THF)
ecFolD	21.31	10.91	31.60
spFolD	42.18	34.21	38.07
hsMTHFD1 DC301	5.93	58.69	20.59
hsMTHFD2	6.42	16.69	12.67

[a] it is nicotinamide adenine dinucleotide (NAD^+) in the case of hsMTHFD2

Supplementary Table 5. Binding kinetic information (k_{on} and k_{off}) and K_D calculated by kinetics or affinity.

Enzyme	k_{on} (1/Ms)	k_{off} (1/s)	K_D (M) (by kinetics)	K_D (M) (by affinity)
ecFolD	1.043×10^{6}	0.01003	9.62×10^{-9}	8.76×10^{-9}
spFolD	1.188×10^{6}	0.03156	26.57×10^{-9}	18.74×10^{-9}
hsMTHFD2	1.170×10^{6}	0.02220	18.98×10^{-9}	14.44×10^{-9}
ecFolD Q98H	5.815×10^{4}	0.6084	1.05×10^{-5}	1.95×10^{-5}

Supplementary Table 6. Data collection and refinement statistics

	Apo-FolD^{Meth}	FolD^{Meth}-caro	FolD^{Meth}Q98H
**Data collection**			
Space group	P1 2_1 1	P1 2_1 1	P1 2_1 1
Cell dimensions			
a, *b*, *c* (Å)	99.6, 79.8, 101.4	99.7, 81.1, 101.0	100.1, 79.4, 101.8
α, β, γ (°)	90.0, 113.3, 90.0	90.0, 112.9, 90.0	90.0, 113.8, 90.0
Resolution (Å)	1.89 (1.94-1.89)	2.10 (2.21-2.10)	1.90 (2.00-1.90)
R_{merge}	8.4 (79.80)	9.1 (60.6)	6.1 (54.1)
$I / \sigma I$	17.9 (2.4)	10.4 (2.5)	22.1 (4.4)
Completeness (%)	99.8 (98.9)	98.9 (99.8)	98.9 (99.2)
Redundancy	6.7 (6.5)	4.4 (4.5)	9.7 (9.9)
**Refinement**			
Resolution (Å)	36.59 - 1.89	45.88 - 2.10	46.56 - 1.90
No. reflections	116,659	85,476	113,624
R_{work} / R_{free}	0.190/0.216	0.175/0.206	0.186/0.198
No. atoms			
Protein	8640	8441	8658
Ligand/ion		132	
Water	1014	588	876
B-factors	36.22	42.23	34.75
Protein	35.90	41.65	33.97
Ligand/ion		69.76	
Water	38.92	44.16	42.51
R.m.s. deviations			
Bond lengths (Å)	0.003	0.005	0.013
Bond angles (°)	0.65	0.70	1.11

Supplementary Table 7. Enzyme kinetic parameters for all FolD enzymes in this study.

enzyme	Dehydrogenase				
	substrate	K_m (μM)	k_{cat} (s^{-1})	k_{cat}/K_m (s^{-1} μM^{-1})	Specific activity (μmol min^{-1} mg^{-1})
ecFolD	5,10-CH_2-THF	76.47 ± 8.27	90.34 ± 3.29	1.18	173.57 ± 6.32
	NADP	186.51 ± 21.02	89.64 ± 3.54	0.48	172.22 ± 6.81
ecFolD G8S	5,10-CH_2-THF	179.44 ± 26.56	20.27 ± 1.75	0.11	35.67 ± 3.08
	NADP	510.80 ± 35.67	20.74 ± 0.71	0.04	36.50 ± 1.25
ecFolD K54N	5,10-CH_2-THF	27.03 ± 1.85	18.19 ± 0.39	0.67	32.06 ± 0.39
	NADP	173.31 ± 21.66	16.00 ± 0.40	0.09	28.20 ± 0.68
ecFolD ΔK54R55	5,10-CH_2-THF	61.75 ± 7.29	0.81 ± 0.03	0.01	1.44 ± 0.06
	NADP	675.63 ± 86.00	1.16 ± 0.06	0.0017	2.06 ± 0.11
ecFolD Q98H	5,10-CH_2-THF	160.57 ± 15.87	8.28 ± 0.34	0.05	14.58 ± 0.59
	NADP	311.53 ± 55.44	6.67 ± 0.45	0.02	11.75 ± 0.79
spFolD	5,10-CH_2-THF	76.52 ± 9.25	68.10 ± 3.11	0.89	118.69 ± 5.42
	NADP	70.33 ± 5.53	62.30 ± 1.02	0.89	108.59 ± 1.77
hsMTHFD1 DC301	5,10-CH_2-THF	5.54 ± 0.66	4.32 ± 0.35	0.78	7.15 ± 0.59
	NADP	27.13 ± 6.55	4.85 ± 0.58	0.18	8.04 ± 0.96
hsMTHFD2	5,10-CH_2-THF	37.29 ± 8.05	15.04 ± 1.14	0.40	25.70 ± 1.95
	NADP	171.67 ± 9.28	33.12 ± 0.43	0.19	28.29 ± 0.74
enzyme	Cyclohydrolase				
	substrate	K_m (μM)	k_{cat} (s^{-1})	k_{cat}/K_m (s^{-1} μM^{-1})	Specific activity (μmol min^{-1} mg^{-1})
ecFolD	5,10-CH=THF	26.63 ± 6.77	155.53 ± 19.71	5.84	298.81 ± 37.87
spFolD	5,10-CH=THF	41.08 ± 8.38	91.33 ± 7.97	2.22	159.17 ± 13.88
hsMTHFD1 DC301	5,10-CH=THF	42.61 ± 9.39	137.56 ± 13.93	3.23	227.79 ± 23.07
hsMTHFD2	5,10-CH=THF	16.14 ± 3.69	242.44 ± 22.41	15.02	414.23 ± 38.29

Supplementary Table 8. The IC_{50} and IC_{80} values determined for carolacton inhibition on different FolD enzymes in this study.

enzyme	carolacton inhibition on DH activity		carolacton inhibition on CYH activity	
	IC_{50} (nM)	IC_{80} (nM)	IC_{50} (nM)	IC_{80} (nM)
ecFolD	15.49	52.22	49.83	180.01
ecFolD G8S	86.49	736.59	ND	ND
ecFolD K54N	ND[a]	ND	ND	ND
ecFolD ΔK54R55	ND	ND	ND	ND
ecFolD Q98H	313.21	1931.66	ND	ND
spFolD	36.80	168.90	38.09	101.34
hsMTHFD1 DC301	38.05	77.58	19.45	67.54
hsMTHFD2	6.50	31.71	85.73	201.78

[a] ND means not determined, because either the enzymatic activity is totally abolished or too low or the enzymatic activity is not affected.

Supplementary Table 9. Plasmids used in this study.

Plasmid	Relevant genotype	Reference
pEHISTEV	kan^R	Liu, 2009[7]
pEHISTEV::*ecfolD*	pEHISTEV, kan^R, $folD_{Eco}$	This work
pEHISTEV::*ecfolD G8S*	pEHISTEV, kan^R, $folD_{EcoG8S}$	This work
pEHISTEV::*ecfolD Q98H*	pEHISTEV, kan^R, $folD_{EcoQ98H}$	This work
pEHISTEV::*ecfolD K54N*	pEHISTEV, kan^R, $folD_{EcoK54N}$	This work
pEHISTEV::*ecfolD ΔK54R55*	pEHISTEV, kan^R, $folD_{Eco\Delta K54R55}$	This work
pEHISTEV::*spfolD*	pEHISTEV, kan^R, $folD_{Spn}$	This work
pEHISTEV::*mthfd1_DC*	pEHISTEV, kan^R, *mthfd1_DC*	This work
pEHISTEV::*mthfd2*	pEHISTEV, kan^R, *mthfd2*	This work

Supplementary Table 10. Oligonucleotides used for constructing FolD expression plasmids.

Target gene	Primer	Sequences (5' to 3')[a]
E. coliΔtolC folD, Q98H, K54N, ΔK54R55 *folD*	ecFolD_NcoI_F	ATACCATGGGCGCAGCAAAGATTATTGACGGTAAAAC
	ecFolD_HindIII_R	GTCAAGCTTTTACTCATCCTGTGGATCATGATAT
E. coliΔtolC G8S *folD*	G8S_NcoI_F	ATACCATGGGCGCAGCAAAGATTATTGACAGTAAAAC
	ecFolD_HindIII_R	GTCAAGCTTTTACTCATCCTGTGGATCATGATAT
S. pneumoniae TIGR4 *folD*	spFolD_EcoRI_F	TCCGAATTCACACAGATTATTGATGGGAAAGCTTTA
	spFolD_XhoI_R	GCACTCGAGTTATTTTCTATCCAATGTCCTAAGTG
Mthfd1[b]	hsMTHFD1_NcoI_F	GCGCCATGGCTCCAGCAGAAATCCTGAA
	hsMTHFD1_HindIII_R	CGCAAGCTTTCACTGAATCATCCACTTTCCT
mthfd2 (without signal peptide region)	hsMTHFD2_EcoRI_F	TCCGAATTCATGGAAGCTGTTGTCATTTCTGGAAG
	hsMTHFD2_HindIII_R	CGCAAGCTTTTAATTAGTGGCTACCCCAA

[a]The underlined characters indicate the restriction sites introduced; [b]only dehydrogenase and cyclohydrolase encoding region

Supplementary Table 11. The buffers for protein purification

<table>
<tr><th>Protein</th><th>Lysis buffer</th><th>Elution buffer</th><th>Desalting buffer</th><th>Gel filtration buffer</th></tr>
<tr><td>ecFolD</td><td rowspan="5">20 mM Bis-Tris pH 6.8
150 mM NaCl
20 mM imidazole</td><td rowspan="5">20 mM Bis-Tris pH 6.8
150 mM NaCl
250 mM imidazole</td><td rowspan="5">20 mM Bis-Tris pH 6.8
150 mM NaCl</td><td>10 mM HEPES
150 mM NaCl
1mM TCEP</td></tr>
<tr><td>ecFolDG8S</td><td></td></tr>
<tr><td>ecFolDQ98H</td><td></td></tr>
<tr><td>ecFolDK54N</td><td></td></tr>
<tr><td>ecFolDΔK54R55</td><td></td></tr>
<tr><td>spFolD</td><td rowspan="2">20 mM Bis-Tris pH 6.8
200 mM NaCl
10% glycerol
20 mM imidazole
1 mM TCEP</td><td rowspan="2">20 mM Bis-Tris pH 6.8
200 mM NaCl
10% glycerol
250 mM imidazole
1 mM TCEP</td><td rowspan="2">20 mM Bis-Tris pH 6.8
200 mM NaCl
10% glycerol
1 mM TCEP</td><td></td></tr>
<tr><td>MTHFD1_DC</td><td rowspan="2">10 mM HEPES
150 mM NaCl
1mM TCEP</td></tr>
<tr><td>MTHFD2</td><td>20 mM Tris-HCl pH 8.0
200 mM NaCl
10% glycerol
20 mM imidazole</td><td>20 mM Tris-HCl pH 8.0
200 mM NaCl
10% glycerol
250 mM imidazole</td><td>20 mM Tris-HCl pH 8.0
200 mM NaCl
10% glycerol</td></tr>
</table>

Supplementary Table 12. Ramachandran statistics for the ecFolD strutcures after final refinement.

	**ecFolD^{Meth}**	**ecFolD^{Meth}-carolacton**	**ecFolD^{Meth}Q98H**
**Ramachandran favored (%)**	95.75	97.14	96.77
**Ramachandran allowed (%)**	3.97	2.77	3.05
**Ramachandran outliers (%)**	0.27	0.09	0.18

Supplementary References

1. Eadsforth, T.C., Maluf, F.V. & Hunter, W.N. Acinetobacter baumannii FolD ligand complexes --potent inhibitors of folate metabolism and a re-evaluation of the structure of LY374571. *FEBS J* **279,** 4350–4360 (2012).
2. D'Ari, L. & Rabinowitz, J.C. Purification, characterization, cloning, and amino acid sequence of the bifunctional enzyme 5,10-methylenetetrahydrofolate dehydrogenase/5,10-methenyltetrahydrofolate cyclohydrolase from Escherichia coli. *J Biol Chem* **266,** 23953–23958 (1991).
3. Dev, I.K. & Harvey, R.J. A complex of N5,N10-methylenetetrahydrofolate dehydrogenase and N5,N10-methenyltetrahydrofolate cyclohydrolase in Escherichia coli. Purification, subunit structure, and allosteric inhibition by N10-formyltetrahydrofolate. *J Biol Chem* **253,** 4245–4253 (1978).
4. Sah, S. & Varshney, U. Impact of Mutating the Key Residues of a Bifunctional 5,10-Methylenetetrahydrofolate Dehydrogenase-Cyclohydrolase from Escherichia coli on Its Activities. *Biochemistry* **54,** 3504–3513 (2015).
5. Wohlfarth, G., Geerligs, G. & Diekert, G. Purification and characterization of NADP(+)-dependent 5,10-methylenetetrahydrofolate dehydrogenase from Peptostreptococcus productus marburg. *J. Bacteriol.* **173,** 1414–1419 (1991).
6. Clark, J.E. & Ljungdahl, L.G. Purification and properties of 5,10-methenyltetrahydrofolate cyclohydrolase from Clostridium formicoaceticum. *J Biol Chem* **257,** 3833–3836 (1982).
7. Liu, H. & Naismith, J.H. A simple and efficient expression and purification system using two newly constructed vectors. *Protein Expr Purif* **63,** 102–111 (2009).
